# Early administration of MPC-n(IVIg) selectively accumulates in ischemic areas to protect inflammation-induced brain damage from ischemic stroke

**DOI:** 10.7150/thno.58947

**Published:** 2021-07-13

**Authors:** Weili Jin, Ye Wu, Ning Chen, Qixue Wang, Yunfei Wang, Yansheng Li, Sidi Li, Xing Han, Eryan Yang, Fei Tong, Jialing Wu, Xubo Yuan, Chunsheng Kang

**Affiliations:** 1 Department of Neurosurgery, Tianjin Medical University General Hospital, Laboratory of Neuro-oncology, Tianjin Neurological Institute, Key Laboratory of Post-Neurotrauma Neuro-Repair and Regeneration in Central Nervous System, Ministry of Education and Tianjin City, Tianjin 300052, China.; 2 Tianjin Key Laboratory of Composite and Functional Materials, School of Materials Science and Engineering, Tianjin University, Tianjin 300072, China.; 3 Tianjin Key Laboratory of Cerebral Vascular and Neurodegenerative Diseases, Tianjin Neurosurgical Institute, Tianjin Huanhu Hospital, Tianjin 300350, China. Department of Neurology, Tianjin Huanhu Hospital, Tianjin 300350, China.; These authors contributed equally: Weili Jin, Ye Wu

**Keywords:** blood-brain barrier, selective accumulation, intravenous immunoglobulin, immunomodulatory therapy, ischemic stroke

## Abstract

Ischemic stroke is an acute and severe neurological disease, which leads to disability and death. Immunomodulatory therapies exert multiple remarkable protective effects during ischemic stroke. However, patients suffering from ischemic stroke do not benefit from immunomodulatory therapies due to the presence of the blood-brain barrier (BBB) and their off-target effects.

**Methods:** We presented a delivery strategy to optimize immunomodulatory therapies by facilitating BBB penetration and selectively delivering intravenous immunoglobulin (IVIg) to ischemic regions using 2-methacryloyloxyethyl phosphorylcholine (MPC)-nanocapsules, MPC-n(IVIg), synthesized using MPC monomers and ethylene glycol dimethyl acrylate (EGDMA) crosslinker via in situ polymerization. *In vitro* and *in vivo* experiments verify the effect and safety of MPC-n(IVIg).

**Results:** MPC-n(IVIg) efficiently crosses the BBB and IVIg selectively accumulates in ischemic areas in a high-affinity choline transporter 1 (ChT1)-overexpression dependent manner via endothelial cells in ischemic areas. Moreover, earlier administration of MPC-n(IVIg) more efficiently deliver IVIg to ischemic areas. Furthermore, the early administration of low-dosage MPC-n(IVIg) decreases neurological deficits and mortality by suppressing stroke-induced inflammation in the middle cerebral artery occlusion model.

**Conclusion:** Our findings indicate a promising strategy to efficiently deliver the therapeutics to the ischemic target brain tissue and lower the effective dose of therapeutic drugs for treating ischemic strokes.

## Introduction

Stroke is the second most frequent cause of death and leading source of permanent disability worldwide 1. Ischemic stroke accounts for 80-90% of all strokes and results in devastating brain damage and severe neurological deficits 2, 3. During ischemic stroke, the sudden cessation of cerebral blood supply in a vascular territory generates an ischemic core, surrounded by a hypoperfused but potentially salvageable region called the ischemic penumbra 4. Minutes after ischemic insult, the death of neurocytes orchestrates an inflammatory response characterized by the activation of focal glia, infiltration of peripheral immune cells, and release of cytokines and chemokines to further damage the brain parenchyma and vasculature 5, 6. In ischemic stroke, complement component 3 (C3) is critical in the complement cascade and immune recognition 7-9. Various clinical trials and experimental studies have demonstrated that inflammation following brain ischemia is pivotal in the pathogenesis of stroke 5, 6, 10. Immune regulation maintains immune homeostasis and promotes the resolution of inflammation, thereby decreasing neurological deficits and improving the outcome of strokes 10. Although various immunomodulatory agents are efficacious anti-inflammatory and immunomodulatory therapeutics for numerous diseases, there are challenges to fully utilizing their therapeutic potential in ischemic stroke owing to their incapacity to adequately cross the blood-brain barrier (BBB) 11, a tightly sealed filter formed by blood vessels that prevents cells, unwanted biomolecules, and drugs from accessing the brain 11, 12. The timing of the inflammatory response is important for the pathogenesis of ischemic stroke and should be considered when designing immunomodulatory therapies 5, 6. Preclinical studies in rodent models suggest that anti-inflammatory and immunomodulatory therapies have an extended therapeutic window and limit ischemic injury as up to 12-24 hours after the onset of stroke 6. Even within this window, earlier treatment leads to better outcomes 6. Therefore, immunomodulatory therapy should be administered as soon as possible to minimize ischemic stroke-induced brain damage 13.

There has been significant progress in the discovery of novel immunomodulatory agents 10. Anti-cytokine therapies neutralize inflammation-related cytokines, such as tumor necrosis factor-alpha (TNF-α) and interleukin 1 beta (IL-1β), during inflammation 6. However, the off-target effects of these therapies may lead to adverse effects involving in unwanted toxicities, and susceptibility to infections, which is a common post-stroke complication 14-16. Immunoglobulins are glycoproteins produced by immune cells and protect the body from pathogens by binding to them or forming an encapsulation 17, 18. Purified polyclonal immunoglobulin G (>98%) from human plasma, called intravenous immunoglobulin (IVIg), has been approved by the Food and Drug Administration at high doses (≥1 g/kg) for the treatment of various inflammatory and autoimmune diseases, such as Kawasaki's disease, immune thrombocytopenia, humoral immunodeficiency, and bone marrow transplantation 19-21. Additionally, IVIg has shown promise in treating ischemic stroke by directly targeting the immune system and neuronal cells 22-25. However, patients are more likely to have adverse reactions, such as thromboembolic events and skin reactions, to IVIg at high doses 19.

Drug delivery research has focused on addressing the limitation related to off-target effects of systemically administered drugs 15. Over the years, nanocarriers have been developed to target therapies to specific tissues 15, and have improved the therapeutic efficiency of biomolecules or drugs by assistance on excellent BBB penetration in the central nervous system (CNS) 15, 26. High-affinity choline transporters (ChTs) on the surface of microvascular endothelial cells transport circulating choline from the blood to the parenchyma 27. In the CNS, choline is converted to the essential neurotransmitter, acetylcholine, and transported via nicotinic acetylcholine receptors (nAChRs) widely expressed on brain capillary endothelial cells 27. We have previously demonstrated that 2-methacryloyloxyethyl phosphorylcholine (MPC), a choline and acetylcholine analogue, interacts with ChTs and nAChRs similar to acetylcholine and choline 28, 29. Choline and acetylcholine analogues on nanocapsules target the choline transporters and acetylcholine receptors enable the delivery of monoclonal antibodies or neurotrophins to the CNS; this is made more efficient by its biocompatibility with enhanced penetration of BBB 28-30.

In this study, we used the MPC monomer and ethylene glycol dimethyl acrylate (EGDMA) crosslinker to synthesize nanocapsules to encapsulate IVIg, MPC-n(IVIg), using in situ polymerization. EGDMA is stable at a neutral pH but degradable in an acidic environment 31. Driven by noncovalent interactions, MPC and EGDMA were enriched around IVIg. The subsequent polymerization grew a thin layer of polymer around the IVIg, forming IVIg nanocapsules. During the early stages of ischemic stroke, facilitated by ChT1, MPC-n(IVIg) efficiently crossed the BBB without affecting the BBB integrity and selectively accumulated in the ischemic region. In addition, the acute ischemic stroke induces a decrease in pH 32, where MPC-n(IVIg) could cross the BBB and selectively accumulate, thereby releasing IVIg to modulate inflammation by targeting the immune system and neuronal cells. Our data demonstrated the promising potential of MPC-n(IVIg) in efficiently crossing the BBB and selectively targeting the ischemic brain to reduce brain damage and minimize mortality.

## Methods

### Materials

EGDMA, ammonium persulfate (APS, Sigma, 98%), N-(3-aminopropyl) methacrylamide (APM), MPC, N,N,N′,N′-tetramethylethylenediamine (TEMED), acrylamide (AAM), deuterium oxide (D_2_O), bovine albumin (BSA), and B27 supplement were purchased from Sigma-Aldrich. IVIg was procured from Instituto Grifols. Penicillin/streptomycin (1%), and trypsin-EDTA (0.25%) were obtained from Thermo Fisher Scientific. All reagents were used without purification.

### Data collection

Transcriptome data of ischemic stroke were downloaded from the Gene Expression Omnibus (https://www.ncbi.nlm.nih.gov/geo/) database and one dataset from GSE58720 was analyzed in this study.

### Synthesis of MPC-nanocapsules

We synthesized MPC-nanocapsules as described previously with some modifications 28, 33. Briefly, IVIg was dissolved in 5% sorbitol (Solarbio, 98%) and encapsulated within MPC-nanocapsules through free radical polymerization. In the synthesis process, 100%, 50%, and 0% MPC are indicative of the molar ratio of MPC to that of MPC and AAM. Nanocapsules with 100% MPC [MPC-n(IVIg)], 50% MPC, and 0% MPC [n(IVIg)] were synthesized using molar ratios of free IVIg (no nano-coated IVIg): APM: MPC: EGDMA = 1: 1,000: 3,000: 500; free IVIg: APM: MPC: AAM: EGDMA = 1: 1,000: 1,500: 1,500: 500; and free IVIg: APM: AAM: EGDMA = 1: 1,000: 3,000: 500; respectively. Radical polymerization was initiated in situ by adding APS (APS:IVIg = 500:1, n/n) and TEMED (TEMED:APS = 2:1, w/w) together at 4 ℃ for 2 h. Subsequently, the solutions were dialyzed against phosphate buffer saline (10 mM PBS, pH=7.4) to remove unreacted monomers and by-products using dialysis bags (MWCO: 8,000-140,000, Solarbio) for 48 h and then passed through a hydrophobic interaction column (Phenyl-Sepharose CL-4B, Solarbio, laboratory reagent) to remove unencapsulated proteins. The purified MPC-nanocapsules were stored at 4 °C until use.

### Determination of the protein nanocapsule concentration and the MPC molar content in MPC-n(IVIg)

The concentrations of the nanocapsules, including MPC-n(BSA), and MPC-nanocapsules with different MPC contents, were determined by the concentration of protein in the nanocapsules 29 using a bicinchoninic acid (BCA) protein assay kit (Solarbio) with a standard curve established as per the manufacturer's instructions. In this study, the dose of nanocapsules was determined by that of proteins in the nanocapsules. Briefly, we mixed 200 μL of BCA Reagent A with 4 μL of BCA Reagent B for preparation. Next, each sample (20 μL) was re-dispersed in 180 μL of prepared solutions. Subsequently, the mixtures were incubated at room temperature for 30 min, and absorbance was measured at 562 nm using a microplate reader.

The MPC molar content in MPC-n(IVIg) were quantified via ^1^H-NMR. MPC-n(IVIg) was dissolved in D_2_O and PEG (PEG-OH, 2000 kDa) was added as internal standard with the final concentration of PEG was 5 mg/mL. Then ^1^H NMR spectra were recorded at room temperature. The molar concentration of MPC was calculated by the peak area ratio of PEG to MPC. The molar concentration of IVIg in the nanocapsules was quantified through the BCA kit. The molar ratio of IVIg to MPC was calculated by the following formula:

the molar ratio (IVIg: MPC) = the molar concentration of IVIg / the molar concentration of MPC in MPC-n(IVIg)

The molar ratio of IVIg to MPC in MPC-n(IVIg) is 1:3677.

### Encapsulation rate and drug loading percentage of IVIg in MPC-n(IVIg)

The encapsulation rate of MPC-n(IVIg) was calculated as follows 34:

Encapsulation rate (%) = X_1_/X_2_×100%

where X_1_ and X_2_ represent the total amount of IVIg in purified and non-purified MPC-n(IVIg). The amount of IVIg was measured using a BCA protein assay kit, as previously described 28, 30. The encapsulation rate of IVIg in MPC-n(IVIg) was 25.1 ± 3.5%.

The drug loading percentage of IVIg was calculated as follows 34:

Drug loading efficiency (wt%) = IVIg mass in MPC-n(IVIg) / MPC-n(IVIg) mass × 100%

MPC-n(IVIg) was dialyzed against distilled water, and then lyophilized to yield the MPC-n(IVIg) mass. Next, MPC-n(IVIg) was dissolved in distilled water, and the concentration of IVIg was measured using a BCA protein assay kit to calculate the IVIg mass. The drug loading percentage of IVIg in MPC-n(IVIg) was 11.6±1.4%.

### Characterization of MPC-nanocapsules

Transmission electron microscopy (TEM) was performed for nanocapsules using the JEM-2100F field emission electron microscope with an acceleration voltage of 200 kV. Moreover, TEM was performed on MPC-n(IVIg) (0.1 mg/mL) using a carbon-coated copper grid followed by negative staining using 2% (w/v) phosphotungstic acid solution (Solarbio). The hydrodynamic sizes and zeta potentials of nanocapsules with 100%, 50%, and 0% MPC in PBS (10 mM, pH=7.4) were studied using dynamic light scattering (DLS) at room temperature. To measure the hydrodynamic size, we performed DLS measurements at 90° on the Zetasizer Nano instrument (Malvern Instruments Ltd., United Kingdom). We resuspended 10 μL of 0.5 mg/mL IVIg or MPC-n(IVIg) sample in 2× loading buffer (10 μL, Solarbio). The final concentration of IVIg or MPC-n(IVIg) was 0.25 mg/mL. Subsequently, these samples were subjected to 10% (w/v) sodium dodecyl sulfate-polyacrylamide gel electrophoresis (SDS-PAGE) at 80V for 150 min followed by staining with Coomassie brilliant blue as per the manufacturer's instructions. MPC, IVIg, and MPC-n(IVIg) were dissolved in D_2_O and proton nuclear magnetic resonance (^1^H NMR) spectra were measured using AVANCE IIITM HD 400 MHz NanoBAY (Bruker). In the ultraviolet-visible (UV-vis) spectroscopy, the characteristic absorption peak of IVIg, a kind of protein, was 280 nm. After purified, unencapsulated IVIg was removed. Therefore, the UV-vis spectroscopy of MPC-n(IVIg) with characteristic peaks at 280 nm was attributed to IVIg encapsulated in nanocapsules, which indicated successful MPC-n(IVIg) synthesis. MPC-n(IVIg) was dispersed in PBS (pH 7.4) and absorbance was measured within the 200-800 nm range using a UV-vis spectrophotometer (AOE instruments, A360 spectrophotometer).

### Release kinetics of MPC-n(IVIg)

As previously described 28, 33, the release kinetics of MPC-n(IVIg) were quantified using an enzyme-linked immunosorbent assay (ELISA) kit. Briefly, purified MPC-n(IVIg) was incubated in PBS (200 μL) at different pH conditions (pH 6.0 and pH 7.4) at 37 °C for 48 h. MPC-n(IVIg) solution at a final concentration of 25 μg/mL was kept at pH 6.5 and 7.4 under unstirred conditions. At selected time intervals, the sample (20 μL) in each group was collected and re-dispersed in a 96-well ELISA plate pretreated with the capture antibody. The samples were submitted to ELISA with a standard curve established as per manufacturer's instructions (Table S1).

### The stability of MPC-n(IVIg) in H_2_O, PBS, Dulbecco's modified Eagle's medium (DMEM), and serum

Purified MPC-n(IVIg) was incubated in H_2_O, PBS, DMEM, and serum at 37 °C for 48 h. At selected time intervals, MPC-n(IVIg) was re-dispersed in H_2_O, PBS, DMEM, and serum. The stability of MPC-n(IVIg) was quantified using the scattering light intensity ratio I/I_0_ by DLS, where I_0_ and I represent the initial and time-dependent size of MPC-n(IVIg), respectively.

### Animals

Eight-to ten-week-old male C57BL/6 mice were bred and housed in a pathogen-free facility equipped with individual ventilation systems, irradiated bedding, and a 12-h light/dark cycle. Primary neuronal cultures were obtained from embryos (E15; mixed gender) from 8-to 12-week-old pregnant female C57BL/6 mice. All animals used in the *in vivo* and *in vitro* experiments were healthy and unused in any previous procedures or treated with any drugs.

### Preparation of mouse primary cortical neurons and cell cultures

Embryonic cerebral cortices were dissected, washed in Hank's balanced salt solution (GIBCO), homogenized in trypsin (0.125%, GIBCO), and DNase I (10 U/mL, Solarbio) for 30 min at 37 ℃ in a 5% CO_2_ incubator. The cells were incubated in minimum essential medium (GIBCO) containing 10% heat-inactivated fetal bovine serum (FBS, HyClone) and 1% penicillin/streptomycin (Thermo Fisher Scientific) and seeded in 96-well plates, 6-well plates, or glass-bottom imaging dishes (NEST) coated with poly-L-lysine (Solarbio) at a density of 10^5^ cells/mL. After 4 h of incubation, the medium was replaced with serum-free neurobasal (GIBCO) with 1% Glutamax (GIBCO) and 1% B27 supplement (Sigma) and the cells were cultured at 37 °C in a 5% CO_2_ incubator. Half of the medium was replaced every three days, and the cells were allowed to mature for 15 d in the presence of 5% CO_2_ and 95% air at 37 ℃ before the experiments were conducted.

RAW 264.7, BV-2, and murine brain endothelial bEND.3 cells were contributed from the Tianjin Key Laboratory of Cerebrovascular and Neurodegenerative Diseases, Tianjin Huanhu Hospital and cultured in the presence of 5% CO_2_ and 95% air at 37 ℃ in DMEM (GIBCO) supplemented with 10% FBS and 1% penicillin/streptomycin. The cell lines were passaged upon reaching 70-80% confluency.

### Oxygen and glucose deprivation /reoxygenation (OGD/R)

OGD/R, a well-established *in vitro* model, mimics the conditions of ischemic/reperfusion damage. Primary cortical neuronal cells were seeded at a density of 1×10^5^ cells/mL and bEND.3, BV-2, and RAW 264.7 cells were seeded in 6-well plates at a density of 2×10^5^ cells/well. First, the cells were incubated in glucose-free DMEM (GIBCO) at 37℃ and saturated with a humidified atmosphere of 1% O_2_, 94% nitrogen, and 5% CO_2_ for OGD. The test formulation was added to the complete medium for preparation. After OGD, the cells were rinsed with PBS three times and subsequently incubated in complete culture medium in the presence of 95% air and 5% CO_2_ for reperfusion. Control cells were subjected to the same washing and medium changes but always maintained in complete culture medium under conditions of 95% air and 5% CO_2_ at 37℃.

### *In vitro* BBB model and transendothelial electrical resistance (TEER)

The *in vitro* BBB model was constructed after a monolayer of bEND.3 cells (10^5^ cells/well) was seeded in the upper chamber of the culture inserts (Corning) that were put into 24-well plates. The cell culture medium was replaced every two days. The BBB models were treated with nanocapsules once the TEER of the bEND.3 cells monolayer reached the maximum plateau impedance value 35. The TEER values of the bEND.3 cell monolayer at 0 h were set as one, and data were expressed as relative values. TEER was measured using the Electrical Cell Impedance Sensing array (ECIS; Applied BioPhysics, America).

### Transport assay

When the TEER value of the monolayer bEND.3 cells was >200 Ω×cm^2^, the nanocapsules with different MPC contents [cyanine 5 (Cy5)-labeled IVIg, 1 mg/mL] were applied to the luminal compartment for 0, 2, 4, 6, 8, 10, 12, and 24 h. Subsequently, 200 μL of the medium was collected from the abluminal compartment. Finally, the penetration ratio (%) of the nanocapsules was measured by determining the fluorescence of the abluminal compartment using a microplate reader.

### Apoptosis

A total of 10^6^ primary cortical neurons were resuspended in a single-cell suspension and washed three times with PBS. Cell apoptosis was analyzed using the Annexin V-FITC/7AAD Apoptosis Detection Kit (BestBio) in accordance with the manufacturer's protocols. The rates of apoptosis were detected by flow cytometry (BD FACSCanto II).

### Cell transfection

bEND.3 cells were seeded into 6-well plates at a density of 2×10^5^ cells/well overnight, followed by transfected with siRNAs (30 pmol/well) prepared by Shanghai Integrated Biotech Solutions Co. Ltd. using Lipofectamine 3000 (5 μL/well, Invitrogen). Transfected cells were further incubated in a humidified atmosphere with 5% CO_2_. After 48 h, ChT1 expression was confirmed by Western blot. Cells transfected with scramble or HIF-1α were subjected to OGD for 12 h. Subsequently, HIF-1α expression was confirmed by Western blot. The indicated antibodies and sequences of siRNAs against specific targets are listed in Table S1 and Table S2, respectively.

### RNA extraction and quantitative real-time polymerase chain reaction (qRT-PCR)

Total RNA was isolated from BV-2 cells, and bEND.3 cells using TRIzol (Invitrogen) in accordance with the manufacturer's protocol. cDNAs were synthesized with a PrimeScript RT reagent kit (TaKaRa) and qRT-PCR was performed using SYBR Green Master Mix (Thermo Fisher). The levels of the indicated genes were normalized to β-actin using the 2^-ΔΔCt^ method. Replicates from a minimum of three independent samples were used for qRT-PCR. The primer sequences used for qRT-PCR in this study are listed in Table S3.

### Cell uptake

Fluorescence-activated cell sorting (FACS) was performed as described previously 28. Briefly, bEND.3 cells were plated in 24-well plates at a density of 2×10^5^ cells/well. After OGD, the cells were washed with PBS three times and incubated with complete culture medium with the same concentration of the Cy5-labeled IVIg or nanocapsules (Cy5-labeled IVIg) at 37°C with 95% air and 5% CO_2_ for 0, 4, 6, and 8 h. The cells were rinsed with PBS three times, harvested, and resuspended in 0.2 mL of 4% paraformaldehyde (PFA, Solarbio) before FACS analysis (BD FACSCanto II).

### Macrophage migration

bEND.3 cells (10^5^ cells/well) were cultured on transwell filters (0.4-μm pore size, Millipore) to achieve a confluent monolayer. Migration assays were performed after measuring the TEER values. Vehicle (1× PBS), free IVIg, and MPC-n(IVIg) were added to the top of the transwell filter to cross the BBB *in vitro* for 24 h and conditioned medium in the lower chamber was collected for the following experiments. RAW 264.7 cells were seeded on transwell filters (8-μm pore size, Millipore) and pretreated with 100 ng/mL of lipopolysaccharide (LPS; Sigma-Aldrich) for 3 h. Next, N-formyl methionyl-leucyl-phenylalanine (fMLP; 10 nM; Sigma-Aldrich) was added to the lower chamber to recruit macrophages. Furthermore, RAW 264.7 cells (5×10^5^ cells/well) were seeded in the upper chamber and the conditioned medium were subsequently diluted and transferred to the upper chamber. Finally, the RAW 264.7 cells were allowed to migrate for 24 h before crystal violet staining (Solarbio).

### Experimental ischemic models (filament MCAO method)

Cerebral ischemia was induced by middle cerebral artery occlusion (MCAO) using the intraluminal filament technique, as previously described 36, 37. The mice were anesthetized with intraperitoneal injection of 60 mg/kg sodium pentobarbital (Sigma) 38. All surgeries were performed under sterile conditions. Under anesthesia, a 2-cm incision was made in the middle of the front neck. The left common carotid artery was isolated and temporarily ligated using 6-0 silk. For MCAO, a silicone rubber-coated monofilament (Doccol Corporation) was inserted into the internal carotid artery through the external carotid artery stump and wedged into the circle of Willis to the origin of the middle cerebral artery. The adequacy of MCAO was confirmed by monitoring the cortical blood flow (CBF) at the onset of the occlusion using a laser Doppler flowmetry probe affixed to the skull (Periflux System 5010, Sweden). After occlusion for 45 min, the occluding filament was withdrawn to allow for reperfusion and incision was sutured using 6-0 surgical sutures. Mice subjected to transient focal cerebral ischemia were randomly divided into groups based on the injection of different formulations at 2 h post-reperfusion. Animals with mean CBF > 25% of the pre-ischemic baseline during occlusion or CBF < 80% of the pre-ischemic baseline within 10 min of reperfusion were excluded.

### Evans blue staining (EB)

The mice were transcardially perfused (*via* the left ventricle) with PBS 6 h post intravenous injection of 2% EB (Sigma-Aldrich) following 24 h post-injection of MPC-n(BSA). The brains were harvested, photographed using a digital camera, fixed in 4% PFA, and sectioned to ~5-8-μm-thick slices. The permeability of EB was observed using the Olympus FluoView 1200 confocal microscope (Olympus) at 680 nm upon excitation at 620 nm. To quantify the amount of EB staining, we collected the ipsilateral hemispheres from each group that were weighed, homogenized in 500 µL of N-dimethylformamide (Sigma-Aldrich), and centrifuged at 10,000 rpm for 30 min at 4 ℃. Next, the supernatants were collected, and absorbance was measured at 620 nm using a microplate reader.

### *In vivo* and *ex vivo* distribution, imaging, and quantification

The distribution of MPC-n(IVIg) (Cy5-labeled IVIg) was imaged using the *in vivo* imaging system (IVIS Lumina II, PerkinElmer, USA) 2, 4, and 24 h post-injection and analyzed using Living Image software version 3.1 (Caliper Life Sciences). Mouse brain tissues (left and right brain tissues) were collected at 2 h and 24 h post-injection of non-labeled free IVIg, n(IVIg), and MPC-n(IVIg) after MCAO surgery, and MPC-n(IVIg) in sham groups. For further quantification, brain tissues were collected and homogenate. The concentration of IVIg in the brain parenchyma was quantified by using an ELISA kit (Table S1).

### Immunofluorescence and immunohistochemistry

Immunofluorescence was performed as previously described 28, 39. Briefly, brain sections and cells were incubated with the indicated primary antibodies (Table S1) overnight at 4 °C, washed three times with PBS, and incubated with the secondary antibodies (Table S1) at room temperature for 1 h. Apoptotic cell death in the penumbra and tissue-cultured cells were assayed using the TUNEL Apoptosis Assay Kit in accordance with the manufacturer's instructions. DNA was stained with DAPI (Sigma) and then imaged under an Olympus FluoView 1200 confocal microscope (Olympus). For immunohistochemistry, the slices were incubated with the indicated antibodies (Table S1), biotinylated secondary antibody, diaminobenzidine (DAB, ZSGB-BIO) staining agents, and hematoxylin. The neuropathologist that reviewed and analyzed the stained slices was blinded to the treatment groups. The proportion of stained cell counts/field was subjected to statistical analysis. All images were processed and analyzed using ImageJ.

### Cytokine quantification by ELISA

Mice subjected to transient focal cerebral ischemia were randomly divided into four groups based on the treatment of vehicle, free IVIg (100 and 500 mg/kg), and MPC-n(IVIg) (100 mg/kg) at 2 h post-reperfusion. Brains were collected, weighed, and homogenized in 0.6 mL of 1% Triton X-100 with a homogenizer (JXFSTPRP-48) after intracardiac perfusion with ice-cold PBS. Subsequently, they were sonicated three times for 30 s on ice, centrifuged at 10,000 rpm for 15 min at 4 °C, followed by supernatant collection. The indicated cytokines were quantified using ELISA kits (Table S1) in accordance with the manufacturer's protocol.

### Flow cytometry

Flow cytometry of immune cells was performed as previously described 40. Treated RAW 267.4 cells were digested using 0.25% trypsin, incubated with 1% BSA, stained with FITC-labeled anti-CD86 for 30 min at room temperature, and fixed with 4% PFA. Treated RAW 267.4 cells stained with 1× PBS for 30 min at room temperature were used as the controls in each group. A minimum of 5,000 events was collected for each sample *in vitro*.

*In vivo*, the brains were processed as aforementioned. Ipsilateral hemispheres were dissociated using 1 mg/mL collagenase and 0.1 mg/mL DNase I into a single-cell suspension in accordance with the manufacturer's instructions. Single-cell suspensions were loaded onto 40-80% Percoll gradients (GE Healthcare) and centrifuged for 40 min (800×g) at room temperature to isolate cell debris and myelin. Subsequently, the cells were stained with fluorescence-conjugated antibodies (surface markers for CD45 and CD11b) and fixed with 4% PFA. The cells stained with fluorescence-conjugated CD45 antibody, fluorescence-conjugated CD11b antibody, or 1× PBS were used as controls. A minimum of 5×10^5^ events was collected for each sample *in vivo*. Fluorescence histograms were immediately recorded by flow cytometry (BD FACSCanto II) and analyzed using Flowjo 10.6.2.

### Determining neurological deficit and infarct volume

Mice neurological deficits were evaluated using a five-point neurological deficit score (0, no deficit; 1, failure to extend the right paw; 2, circling to the right; 3, falling to the right; and 4, unable to walk spontaneously) in a blinded fashion 41. Infarct volume was calculated upon sacrificing the mice after evaluating the last neurologic deficit score. Next, the brains were collected, sectioned into 2-mm-thick coronal slices, incubated in 2% 2,3,5-triphenyltetrazolium chloride (TTC, Sigma) at 37℃ for 30 min, and fixed in 4% PFA for 24 h. Brain sections were photographed and analyzed using ImageJ for the infarction volume measurement. To avoid error caused by edema, the percentage of infarct volume was calculated using the following formula: Infarct volume (%) = [contralateral volume (mm^3^) - ipsilateral non-infarct volume (mm^3^)]/[2×contralateral volume (mm^3^)]×100% 42. The infarct volume was examined using three independent brains/treatment group.

### Corner turn test

The mice were allowed to proceed into a 30° corner and freely turn either left or right to exit the corner. The choice of direction during ten trials was recorded and percentage of right turns was calculated. The laterality index (percentage of right turns) was calculated as follows: (number of right turns-number of left turns)/10.

### Statistical analysis

Student's *t*-test was used to compare data between two groups. Gene ontology (GO) analysis was performed using DAVID (http://david.abcc.ncifcrf.gov/). Survival probability was calculated using Kaplan-Meier survival plots and log-rank test. Statistical analyses were performed using GraphPad Prism 7 and Microsoft Excel 2019. Statistical significance was set at P < 0.05.

## Results

### Synthesis and characterization of MPC-n(IVIg)

**Figure 1:**
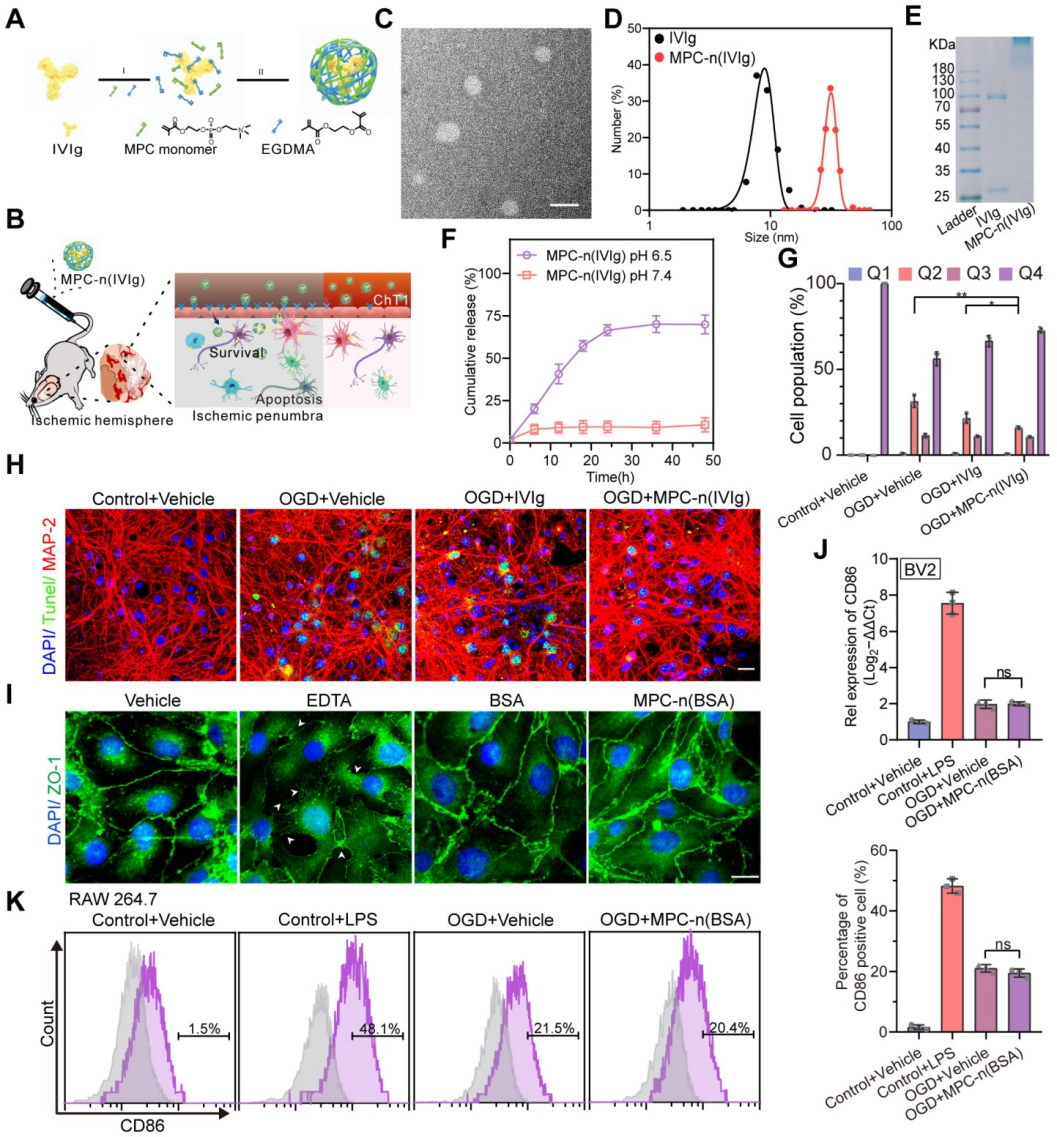
**Characterization, release kinetics, and activity of MPC-nanocapsules. A)** Schematic for the synthesis of MPC-n(IVIg). **B)** Schematic illustration and delivery process of MPC-n(IVIg) that protects against ischemic stroke via intravenous injection by enhancing selective accumulation of IVIg in the ischemic brain region. **C)** Representative transmission electron micrographs of MPC-n(IVIg). Scale bar = 50 nm. **D)** Size distribution of free IVIg and MPC-n(IVIg) measured by dynamic light scattering. The polydispersity index (PDI) of MPC-n(IVIg) is 0.276. **E)** Gel electrophoresis of free IVIg and MPC-n(IVIg). **F)** Release of IVIg from MPC-n(IVIg) in phosphate-buffered saline of different pH (6.5 and 7.4). **G)** Flow cytometry using the Annexin V-FITC/7AAD Apoptosis Detection kit of mature primary cortical neurons subjected to 12 h of oxygen and glucose deprivation (OGD) and subsequently co-incubated with vehicle, free IVIg, or MPC-n(IVIg) (10 mg/mL) for 24 h post-reperfusion (n = 3). **H)** Fluorescence micrographs of TUNEL-stained cells showing apoptosis of mature primary cortical neurons upon OGD for 12 h, followed by incubation with vehicle, free IVIg, or MPC-n(IVIg) (10 mg/mL) for 24 h post-reperfusion. Mature primary cortical neurons incubated with vehicle under normoxic conditions were used as the control. DAPI (Blue), TUNEL (Green), and MAP-2 (Red). Scale bar = 20 μm. **I)** Immunofluorescence of bEND.3 cells treated with vehicle, EDTA, BSA, or MPC-n(BSA) (10 mg/mL) for 24 h to determine the BBB integrity based on staining of the tight junctions targeting ZO-1. Scale bar = 20 μm. **J)** Quantitative reverse transcription-polymerase chain reaction (qRT-PCR) for the mRNA levels of CD86 in BV-2 cells incubated with lipopolysaccharide, IVIg, or MPC-n(IVIg) (10 mg/mL) for 24 h (n = 3). **K)** RAW 264.7 cells were subjected to OGD for 12 h and co-cultured with MPC-n(BSA) (10 mg/mL) for 24 h post-reperfusion (n = 3). CD86 expression was analyzed using fluorescence-activated cell sorting. All experiments were repeated as independent triplicates. All data are presented as the mean ± S.D. ns, nonsignificant, *P < 0.05, **P < 0.01, or ***P < 0.001.

To encapsulate IVIg in nanocapsules and develop an effective strategy for its delivery to the ischemic penumbra, we constructed pH-sensitive MPC-nanocapsules using *in situ* polymerization of MPC monomers with EGDMA as the degradable crosslinker (Figure 1A-B). TEM showed a spherical shape of MPC-n(IVIg) with an average diameter of 31.4 (±2.6) nm, which was further measured by DLS (Figure 1C-D). During electrophoresis, MPC-n(IVIg) was retained in the stacking gel (Figure 1E), which indicated the successful encapsulation of IVIg in MPC-nanocapsules and was consistent with the results of the UV-vis spectroscopy (Figure S1A). Compared with IVIg, the Fourier transform infrared (FT-IR) spectrum of MPC-n(IVIg) exhibited new peaks at 1725 (COO-), 1241 (P=O), 1073 (P-O-C), and 966 (C-N) cm^-1^, which were consisted with the characteristic peaks of MPC 33, and polymer gels without IVIg, indicating the IVIg was covered by the MPC-based polymer shell (Figure S1B). In the ^1^H NMR spectra, the main proton peaks of MPC-n(IVIg) were located at 4.1-4.3, 3.1, and 1.8 ppm, which corresponded to the characteristic peaks of poly 2-methacryloyloxy ethyl phosphorylcholine (PMPC) 33. Moreover, compared with MPC, the proton peaks located at 5.6 and 6.1 ppm (C=CH_2_) were absent in MPC-n(IVIg), indicating the successful synthesis of polymer shell with PMPC as the main component (Figure S1C). These findings confirmed the presence of an MPC-based polymer network in the MPC-n(IVIg). Next, we addressed whether MPC-n(IVIg) was responsive to the acidic environments to release IVIg *in vitro*. We quantified the time-course release of IVIg from the MPC-n(IVIg) under different pH conditions (7.4 and 6.5) at 37°C using ELISA (Figure 1F). MPC-n(IVIg) rapidly released IVIg in the first 24 h at a pH of 6.5. To investigate the stability of MPC-n(IVIg), MPC-n(IVIg) was dissolved in H_2_O, PBS, DMEM, and serum at 37°C, and the size of MPC-n(IVIg) was measured by DLS for 48 h. As shown in Figure S1D, the MPC-n(IVIg) remained stable in H_2_O, PBS, DMEM, and serum at 37°C for 48 h. These findings suggested that the encapsulation of IVIg enabled controlled release and enhanced its stability. IVIg targets the intrinsic neuronal complement cascade to prevent apoptosis 22; thus, we evaluated the activity of IVIg released from MPC-n(IVIg) and apoptosis in mature primary cortical neurons using flow cytometry and terminal deoxynucleotidyl transferase dUTP nick end labeling (TUNEL) analysis. We used a similar concentration of IVIg (10 mg/mL) in the MPC-n(IVIg) group as that used by Tha-In et al. 23, 43 and during *in vitro* research on the immunomodulatory effects of IVIg 20. We investigated the activity of therapeutics from the luminal to the abluminal side of murine BBB transwell *in vitro* models (double-coculture system) 35. There was decreased apoptosis in mature primary cortical neurons subjected to OGD and incubated with MPC-n(IVIg) after reoxygenation for 24 h compared with that in other groups (Figure S2A-C, Figure 1G-H). This suggested that IVIg released from MPC-n(IVIg) maintained its biological activity and effectively protected primary cortical neurons from OGD-induced neuron damage *in vitro.*

To evaluate whether MPC-nanocapsules affected the cell viability and BBB integrity, bEnd.3 cells and mature primary cortical neurons were challenged for 24 h with MPC-n(bovine serum albumin [BSA]) (0-20 mg/mL) after OGD for 12 h. A Cell Counting Kit-8 (CCK8) showed that the different concentrations of MPC-n(BSA) were non-toxic (Figure S3A). Next, we investigated the *in vitro* concentration-dependent impact of MPC-n(BSA) on the BBB permeability. There was no significant impact on the BBB permeability at 24 h using MPC-n(BSA) (10 and 20 mg/mL; Figure S3B-C). Zona occludens 1 (ZO-1) staining, marker of tight junctions, showed an intact and tight monolayer of bEnd.3 cells treated for 24 h with MPC-n(BSA) (10 mg/mL), suggesting that there was no loss of cell-cell contact in the MPC-nanocapsule-treated endothelial cell barrier (Figure 1I). *In vivo,* the BBB permeability was analyzed using classical EB staining (Figure S3D), and the mice were intravenously injected with MPC-n(BSA) (100 mg/kg) at 2 h post-reperfusion: EB could not penetrate through the intact BBB to stain brain tissue in the sham groups and there was no significant difference in MCAO groups. These results demonstrated that MPC-nanocapsules had less effect on the disruption of BBB integrity *in vitro* and *in vivo*. The mice were intravenously injected with MPC-n(BSA) (0, 5, 10, 50, and 100 mg/kg) at 2 h post-reperfusion and euthanized following one intravenous injection of MPC-n(BSA) for 30 d. The main organs (heart, liver, spleen, lung, and kidney) were stained with hematoxylin and eosin (H&E) for histological analysis (Figure S4). H&E staining revealed no significant chronic pathological toxicity and adverse effects. Next, we explored whether the MPC-nanocapsules induced inflammatory responses. Pro-OGD-treated microglia cells (BV-2) incubated with vehicle (1× PBS) and MPC-n(BSA) (10 mg/mL) for 24 h after reoxygenation showed similar mRNA levels of the pro-inflammatory marker CD86 (Figure 1J), which were consistent with the FACS results for resting macrophages (RAW 267.4 cells; Figure 1K). We performed immunohistochemistry to investigate the *in vivo* activation of inflammation in microglia and astrocytes using ionized calcium-binding adapter molecule 1 (Iba1, a biomarker of microglia) and glial fibrillary acidic proteins (GFAP, a biomarker of astrocytes) after treatment with MPC-n(BSA) for 24 h. Compared to the vehicle-treated groups, Iba-1 or GFAP levels remained unchanged in the brains of the sham or MCAO groups that were intravenously injected with MPC-n(BSA) (100 mg/kg) (Figure S5A-D). Thus, MPC-nanocapsules did not induce an inflammatory response owing to their excellent biocompatibility.

### MPC-n(IVIg) selectively accumulates in ischemic areas

**Figure 2:**
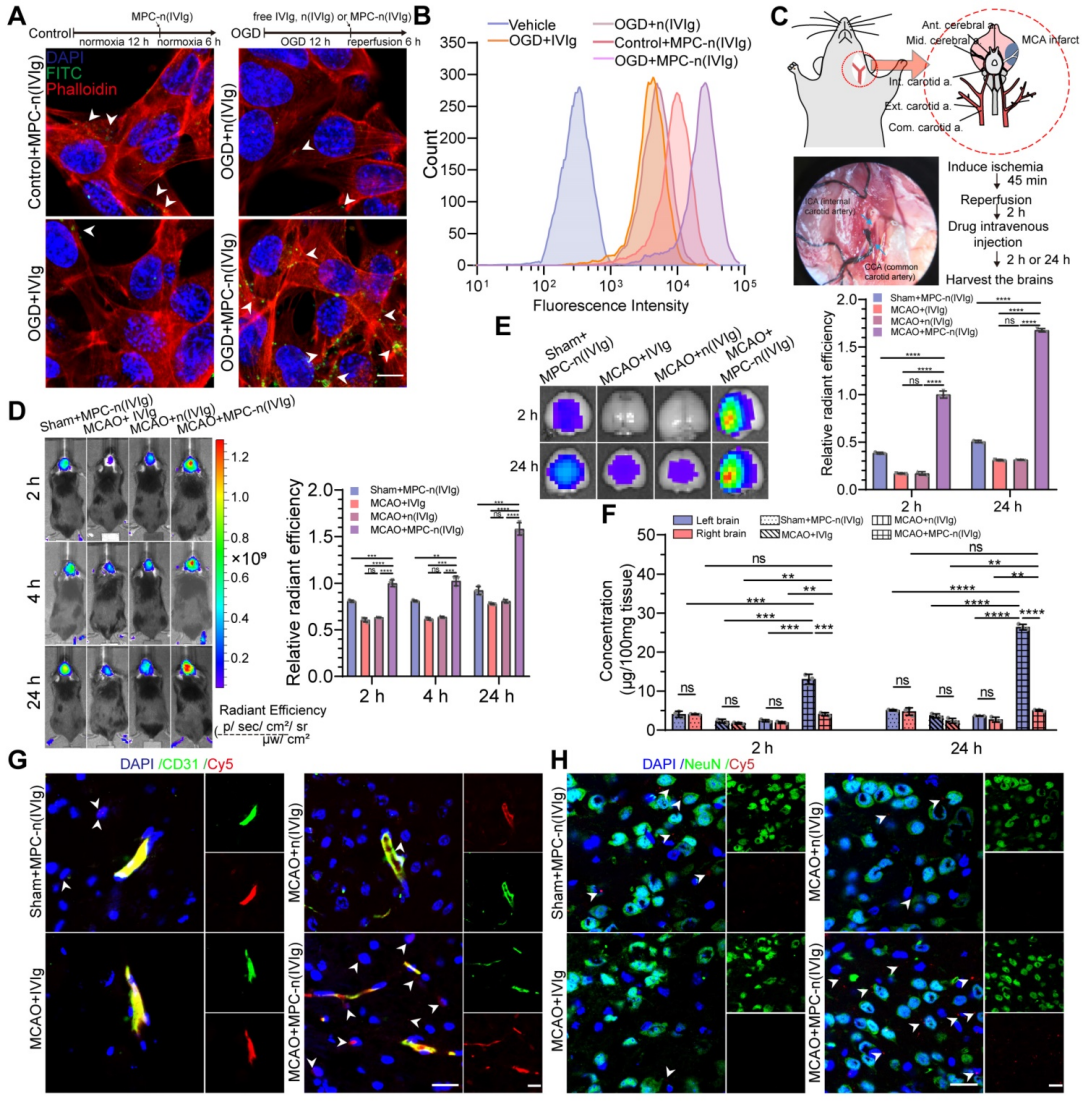
** Selective accumulation of MPC-n(IVIg) in the ischemic regions. A, B)** bEND.3 cells subjected to OGD for 12 h and incubated with vehicle, free IVIg or MPC-n(IVIg) (1 mg/mL) for 6 h post-reperfusion, and bEND.3 cells without OGD for 12 h in control group, but incubated with MPC-n(IVIg) (1 mg/mL) for 6 h under normoxic conditions. Immunofluorescence and flow cytometry showed the uptake of free IVIg, n(IVIg), or MPC-n(IVIg) (1 mg/mL) by bEND.3 cells. Scale bar = 20 μm. **C)** MCAO mice were established under anesthesia using the intraluminal filament method. **D)** Representative images after* in vivo* whole-animal imaging of Cy5 signal at 2, 4, and 24 h after intravenous injection of free IVIg, n(IVIg), or MPC-n(IVIg) (10 mg/kg) in MCAO mice 2 h post-ischemia-reperfusion or that of MPC-n(IVIg) in sham mice. The histogram summarizes the relative radiant efficiency of the brain tissue (n = 3). **E)** Representative* ex vivo* Cy5 signal images of the isolated brain tissues from the treated mice 2 h and 24 h post-injection. The histogram summarizes the relative radiant efficiency of the isolated brain tissue (n = 3). **F)** The concentration of IVIg in the cerebral hemispheres from the treated mice 2 h and 24 h post-injection as measured using enzyme-linked immunosorbent assay (ELISA, n = 3). **G)** Immunofluorescence showing the colocalization of Cy5-labeled IVIg, vascular endothelial cells (CD31), and DAPI-stained nuclei in the brain tissue of the treated mice 24 h post-injection. Scale bar = 20 μm. **H)** Immunofluorescence for the colocalization of Cy5-labeled IVIg, neurons (NeuN), and DAPI-stained nuclei in the brain tissue from the treated mice 24 h post-injection. Scale bar = 20 μm. All experiments were performed as independent triplicates. All data are presented as the mean ± S.D. ns, nonsignificant, *P < 0.05, **P < 0.01, or ***P < 0.001.

Due to the stability and efficient release of MPC-n(IVIg), we evaluated whether MPC nanocapsules enabled the effective delivery of IVIg to ischemic areas. Series nanocapsules with different contents of the MPC were synthesized by using neutral monomer, AAM, to replace 0%, 50%, and 100% of the MPC monomer, respectively. Such nanocapsules showed similar sizes and surface charges (Figure S6A-B). Figure S6C revealed that, compared to nanocapsules with 50% MPC, nanocapsules with 100% MPC showed stronger penetration to cross the bEND.3 layer. Moreover, bEND.3 cells treated with nanocapsules with 100% MPC for 6 h showed the strongest ability of cellular uptake (Figure S6D). Nanocapsules with 100% MPC exhibited the strongest signal in the MCAO models using the IVIS (Figure S6E). These findings validated that MPC endowed MPC-n(IVIg) with the ability to cross the BBB. Next, we validated the effectiveness of delivering IVIg to target the ischemic areas by determining the cellular uptake of MPC-n(IVIg) by bEND.3 cells. Pro-OGD-treated bEND.3 cells (pbEND.3) incubated with fluorescein isothiocyanate (FITC)-labeled MPC-n(IVIg) showed a stronger green fluorescence signal than control bEND.3 cells (Figure 2A).

However, a much weaker fluorescence signal was visible in pbEND.3 cells incubated with FITC-labeled free IVIg and FITC-labeled n(IVIg) (nanocapsules without MPC). Flow cytometry showed that pbEND.3 cells incubated with Cy5-labeled MPC-n(IVIg) had a 2.65-fold higher mean fluorescence intensity than control bEND.3 cells. pbEND.3 cells incubated with MPC-n(IVIg) consistently exhibited a 6.02- and 5.34-fold higher mean fluorescence intensity than those incubated with Cy5-labeled free IVIg and n(IVIg), respectively (Figure 2B). Subsequently, we investigated the duration and ability to target the ischemic hemisphere by MPC-n(IVIg) in mice using biodistribution studies via the tail vein (Figure 2C). After occlusion for 45 min, the occluding filament was withdrawn to enable reperfusion. The mice were intravenously injected 2 h post-reperfusion with nanocapsules. We used the IVIS to assess the accumulation and biodistribution of MPC-n(IVIg) (10 mg/kg of body weight) in the ischemic hemisphere of the MCAO models. Compared to free IVIg and n(IVIg), MPC-n(IVIg) exhibited a stronger signal in the MCAO models 2 h post-injection that was maintained for up to 24 h (Figure 2D, Figure S7A). Consistent with the *in vitro* results, the accumulation of MPC-n(IVIg) was enhanced during ischemic stroke. To further study accumulation in the ischemic hemisphere (damage from MCAO surgery), we transcardially perfused the mice and harvested their brain at 2 h and 24 h post-injection. Administration of free IVIg and n(IVIg) exhibited an intense Cy5 signal in the livers and kidneys that were collected 24 h post-injection, suggesting rapid hepatic and renal clearance (Figure S7B). However, the ipsilateral hemisphere had a stronger Cy5 signal upon administering MPC-n(IVIg) in the MCAO mice, indicating the selective accumulation of MPC-n(IVIg) in the ischemic hemisphere (Figure 2E). Furthermore, we harvested brains from sham and MCAO models at 2 h and 24 h post-injection with non-fluorescent labeled free IVIg, n(IVIg), and MPC-n(IVIg). Subsequently, we separately homogenized the right brain (no damage from MCAO surgery) and left brain (damage from MCAO surgery), and quantitatively measured the accumulation of IVIg using ELISA (Figure 2F). Administration of MPC-n(IVIg) in the left hemisphere exhibited a 3.22-fold and 5.17-fold higher accumulation in the MCAO models than that in the sham models at 2 h and 24 h post-injection, respectively. Moreover, in the right hemisphere, the accumulation of IVIg after administration of MPC-n(IVIg) was more than that of free IVIg and n(IVIg) in MCAO models at 2 h and 24 h post-injection, respectively. These demonstrated that MPC-n(IVIg) had the ability to enhance penetration for normal mouse BBB, which were consisted with our previous studies 28, 29. In contrast, there was no significant difference in the accumulation of IVIg after the administration of MPC-n(IVIg) in the right hemisphere of the MCAO and sham models. There was also no significant difference in the accumulation of IVIg between the left and right hemispheres in the groups excepting the MCAO models injected with MPC-n(IVIg) at 2 h and 24 h post-injection. The accumulation of IVIg in the left hemisphere did not decrease after 24 h in the MCAO model injected with MPC-n(IVIg). This indicated that MPC-n(IVIg) sustained in the ischemic brain tissue for several hours at least. Furthermore, immunofluorescence of the mice brain sections 24 h post-injection of the nanocapsules confirmed that MPC-n(IVIg) crossed the BBB to the ischemic brain parenchyma (Figure 2G-H). These results demonstrated that MPC-nanocapsules not only enhanced the BBB penetration, but promoted the selective accumulation of IVIg in ischemic areas.

### MPC-n(IVIg) selectively targets ischemic areas in a ChT1-dependent manner

**Figure 3:**
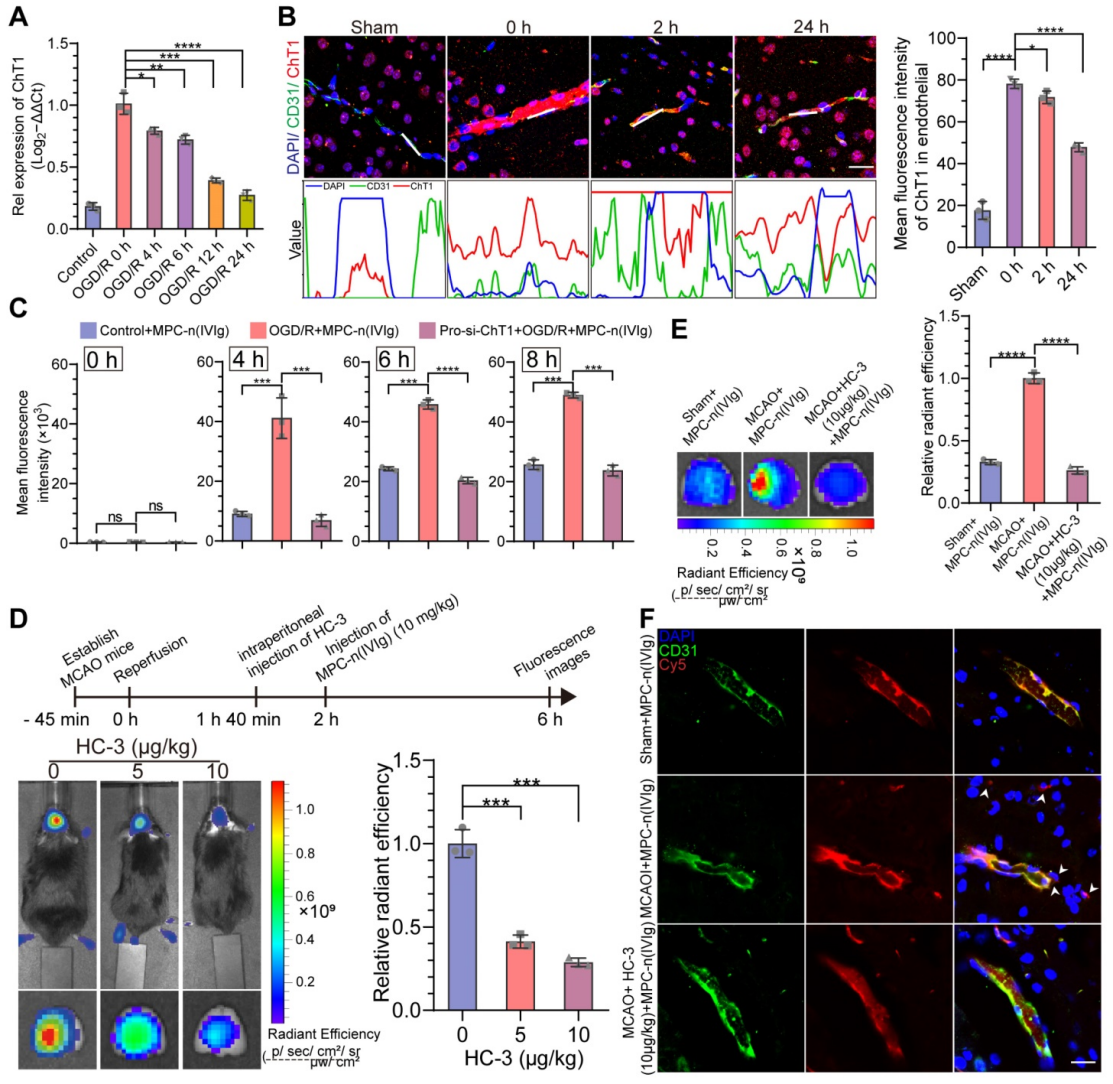
** MPC-n(IVIg) selectively targets ischemic areas in a ChT1-dependent manner in endothelial cells during ischemic stroke. A)** qRT-PCR analysis for the mRNA levels of ChT1 in bEND.3 cells subjected to OGD for 12 h and reperfused for 0, 4, 6, 12, and 24 h (n = 3). **B)** Immunofluorescence for the localization of ChT1 and CD31 on the endothelial cell surface in MCAO mice. The gray values are shown in the line plots. Scale bar = 20 μm. The histogram summarizes the mean fluorescence intensity of ChT1 (n = 3). **C)** Flow cytometry for the uptake of MPC-n(IVIg) by bEND.3 cells following incubation with MPC-n(IVIg) (1 mg/mL) for 0, 4, 6, and 8 h with reperfusion in the control+MPC-n(IVIg), OGD+MPC-n(IVIg), and pro-si-ChT1+OGD+MPC-n(IVIg) groups (n = 3). **D)** *In vivo* whole C57 mice imaging of Cy5 signal 4 h post intravenous injection with MPC-n(IVIg) (10 mg/kg) at 2 h post-ischemia-reperfusion in MCAO mice intraperitoneally preinjected with different doses of HC-3 20 min prior to the injection of MPC-n(IVIg). *Ex vivo* Cy5 signal images of the isolated brain tissue from the treated mice 4 h post-injection. The histogram summarizes the relative radiant efficiency of the isolated brain tissue (n = 3). **E)** *Ex vivo* Cy5 images of the isolated brain tissue from treated mice [sham+MPC-n(IVIg), MCAO+MPC-n(IVIg), and MCAO+HC-3 (10 μg/kg)+MPC-n(IVIg)] 24 h post-injection. The histogram summarizes the relative radiant efficiency of the brain tissue (n = 3). **F)** Immunofluorescence for the colocalization of Cy5-labeled IVIg, CD31, and DAPI-stained nuclei in the brain tissue from the treated mice 24 h post-injection. Scale bar = 20 μm. All experiments were repeated independently three times. All data are presented as the mean ± S.D. ns, nonsignificant, *P < 0.05, **P < 0.01, or ***P < 0.001.

Although acute ischemic injury transiently disrupts the BBB 44, only a small proportion of free IVIg or n(IVIg) accumulated in the ischemic hemisphere. Therefore, there could be limited initial accumulation of free IVIg, n(IVIg), and MPC-n(IVIg) at the ischemic-reperfusion site allowed by BBB disruption induced by ischemic stroke and surgical procedures. Nevertheless, effective BBB penetration is necessary for agents to effectively enter the ischemic hemisphere. The mechanism underlying the selective targeting of MPC-n(IVIg) to the ischemic region needs to be investigated as the direct entry of MPC-n(IVIg) through the disrupted BBB in ischemic regions is not the major route of access to the brain. We have previously reported that the BBB penetration of MPC-nanocapsules that contain choline and acetylcholine analogs is attributed to ChT1-mediated transcytosis 28, 30. We organized the ribonucleic acid (RNA)-array data into biologically coherent networks, applied a difference analysis [-log10(false discovery rate, FDR) >2, fold change>1], and found differentially expressed genes listed in the volcano plot representing the sham and MCAO groups (Figure S8A). Subsequently, we analyzed the GO for the molecular functions of the genes. The upregulated genes were involved in the membrane, transporter complex, and membrane raft (Figure S8B). The mRNA levels of ChT1 in OGD bEND.3 cells increased *in vitro* (Figure S9A). Furthermore, we used Western blot to verify the effect of RNA interference (Figure S9B-C). The overexpression of ChT1 was dependent on HIF-1α upon RNA interference in bEND.3 cells (Figure S9D). However, with prolonged reperfusion, the expression of ChT1 gradually decreased in bEND.3 cells (Figure 3A, Figure S9E). Next, we performed immunofluorescence using the coronal sections from MCAO models at different durations of ischemia-reperfusion. Using a vascular endothelial cell marker (CD31), we further demonstrated that ChT1 was highly expressed in the endothelial cells after ischemic stroke. However, prolonged reperfusion gradually decreased the expression of ChT1 in endothelial cells, consistent with the *in vitro* results (Figure 3B). These data confirmed the overexpression of ChT1 in the endothelia during ischemia; reperfusion decreased ChT1 expression. The cellular uptake of MPC-n(IVIg) by bEND.3 cells with different treatments was investigated to evaluate whether the overexpression of ChT1 facilitated the uptake of MPC-n(IVIg) in endothelial cells during ischemic stroke. Strong green fluorescence was intracellularly observed within the pbEND.3 cells when treated with FITC-labeled MPC-n(IVIg) (Figure S10A). A much weaker fluorescence was visible in the control bEND.3 and pro-si-ChT1-treated pbEND.3 cells (ppbEND.3), which were transfected with siRNA for ChT1 for 48 h, and subsequently subjected to OGD for 12 h. Flow cytometry of the bEND.3 cells co-cultured with Cy5-labeled MPC-n(IVIg) were highly consistent with immunofluorescence (Figure 3C, Figure S10B). Moreover, pbEND.3 cells incubated with MPC-n(IVIg) displayed a 3.27-4.71 and 3.76-8.21-fold higher positive fluorescence signal ratio than the control bEND.3 and ppbEND.3 cells at 4 h post-incubation, respectively. MCAO models were intraperitoneally injected with 0, 5, and 10 μg/kg of the ChT1 inhibitor, hemicholinium-3 (HC-3), 20 mins prior to the injection with Cy5-labeled MPC-n(IVIg). We performed *in vivo* and *ex vivo* Cy5 signal images of the mice brains 4 h after the injection (Figure 3D). Increasing doses of HC-3 reduced Cy5 signal in the ischemic brain, confirming that ChT1 mediated the transport of Cy5-labeled MPC-n(IVIg) into the brain. Moreover, enhanced Cy5 signal was observed in mice at 2, 4, and 24 h after the administration of MPC-n(IVIg) in the MCAO models compared to that in the sham models and MCAO models with intraperitoneally pro-injected 10 μg/kg of HC-3 (Figure S11). This was confirmed using *ex vivo* imaging of the brain harvested from the mice 24 h post-injection (Figure 3E). Immunofluorescence of the brain sections showed more accumulations of MPC-n(IVIg) in the MCAO brain parenchyma compared to the sham brain and MCAO brain injected with HC-3 at 24 h post-injection (Figure 3F). These findings validated that during early ischemia-reperfusion, the overexpression of ChT1 in the ischemic hemisphere could facilitate an efficient delivery strategy for BBB penetration and selective accumulation of MPC-n(IVIg) during ischemic stroke.

### Early administration of MPC-n(IVIg) facilitates IVIg accumulation in ischemic areas

**Figure 4:**
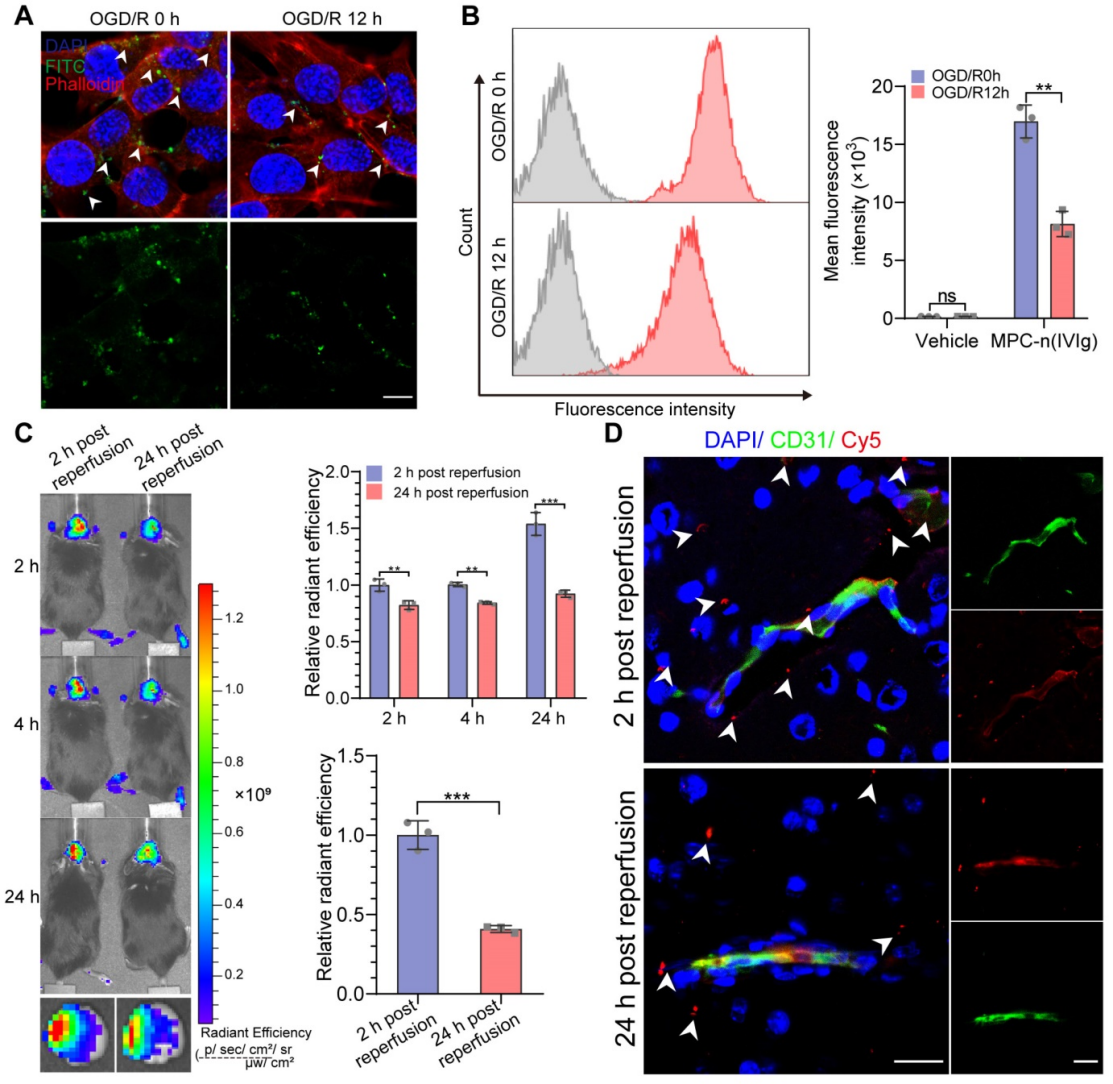
** Early administration of MPC-n(IVIg) facilitates IVIg accumulation in ischemic areas. A, B)** Immunofluorescence and flow cytometry showing the uptake of MPC-n(IVIg) (0.5 mg/mL) by bEND.3 cells following incubation with MPC-n(IVIg) for 6 h after 12 h of OGD with or without reperfusion for 12 h. The histograms summarize the fluorescence intensity of MPC-n(IVIg) in the bEND.3 cells using flow cytometry (n = 3). Scale bar = 20 μm. **C)*** In vivo* whole C57 mice imaging of Cy5 signal at 2, 4, or 24 h post intravenous injection with MPC-n(IVIg) (10 mg/kg) 2 or 24 h after ischemia-reperfusion. *Ex vivo* Cy5 signal images of the isolated brain tissue from the treated mice 24 h post-injection. The histograms summarize the relative radiant efficiency of the whole C57 mice and isolated brain tissue (n = 3). **D)** Immunofluorescence for the colocalization of Cy5-labeled IVIg, CD31, and DAPI-stained nuclei in the brain tissue from the treated mice 24 h post-injection. Scale bar = 20 μm. All experiments were repeated independently three times. All data are presented as the mean ± S.D. ns, nonsignificant, *P < 0.05, **P < 0.01, or ***P < 0.001.

We investigated the time-dependent cellular uptake of MPC-n(IVIg) after reperfusion to evaluate the timing for the administration of MPC-n(IVIg) during ischemic stroke. Due to the overexpression of ChT1 in the cellular membrane during ischemia, the uptake of MPC-n(IVIg) in bEND.3 cells subjected to OGD for 12 h was confirmed by immunofluorescence and flow cytometry after reoxygenation for 0 or 12 h (OGD/R 0 h or OGD/R 12 h). bEND.3 cells incubated with FITC-labeled MPC-n(IVIg) for 4 h showed strong green fluorescence in the OGD/R 0 h group (Figure 4A). Flow cytometry data of the bEND.3 cells co-incubated with Cy5-labeled MPC-n(IVIg) for 4 h was highly consistent with immunofluorescence (Figure 4B). The bEND.3 cells in the OGD/R 0 h group incubated with MPC-n(IVIg) showed a 1.65-2.51-fold higher mean fluorescence intensity than that in the OGD/R 12 h group. Higher accumulation of MPC-n(IVIg) was found in the ischemic brain when administered after 2 h of reperfusion than that after 24 h. *Ex vivo* imaging of the brain and a quantitative analysis revealed that IVIg showed a 2.45-fold increase in the ischemic hemisphere 24 h post-administration of MPC-n(IVIg) after 2 h of reperfusion than that after 24 h (Figure 4C). Immunofluorescence of the brain sections showed that more accumulation of IVIg in the brain parenchyma after 2 h of reperfusion than that after 24 h (Figure 4D). These findings confirmed that early intervention promoted the cellular uptake of MPC-n(IVIg) that could be attributed to the decreased expression of ChT1 in endothelial cells after reperfusion, and indicated that the early administration of MPC-n(IVIg) enhanced the BBB penetration and facilitated the selective accumulation of IVIg in ischemic areas, thereby preventing the progression of stroke-induced damage.

### Low-dosage MPC-n(IVIg) increases neuroprotective effects and reduces ischemic stroke-induced injury

**Figure 5:**
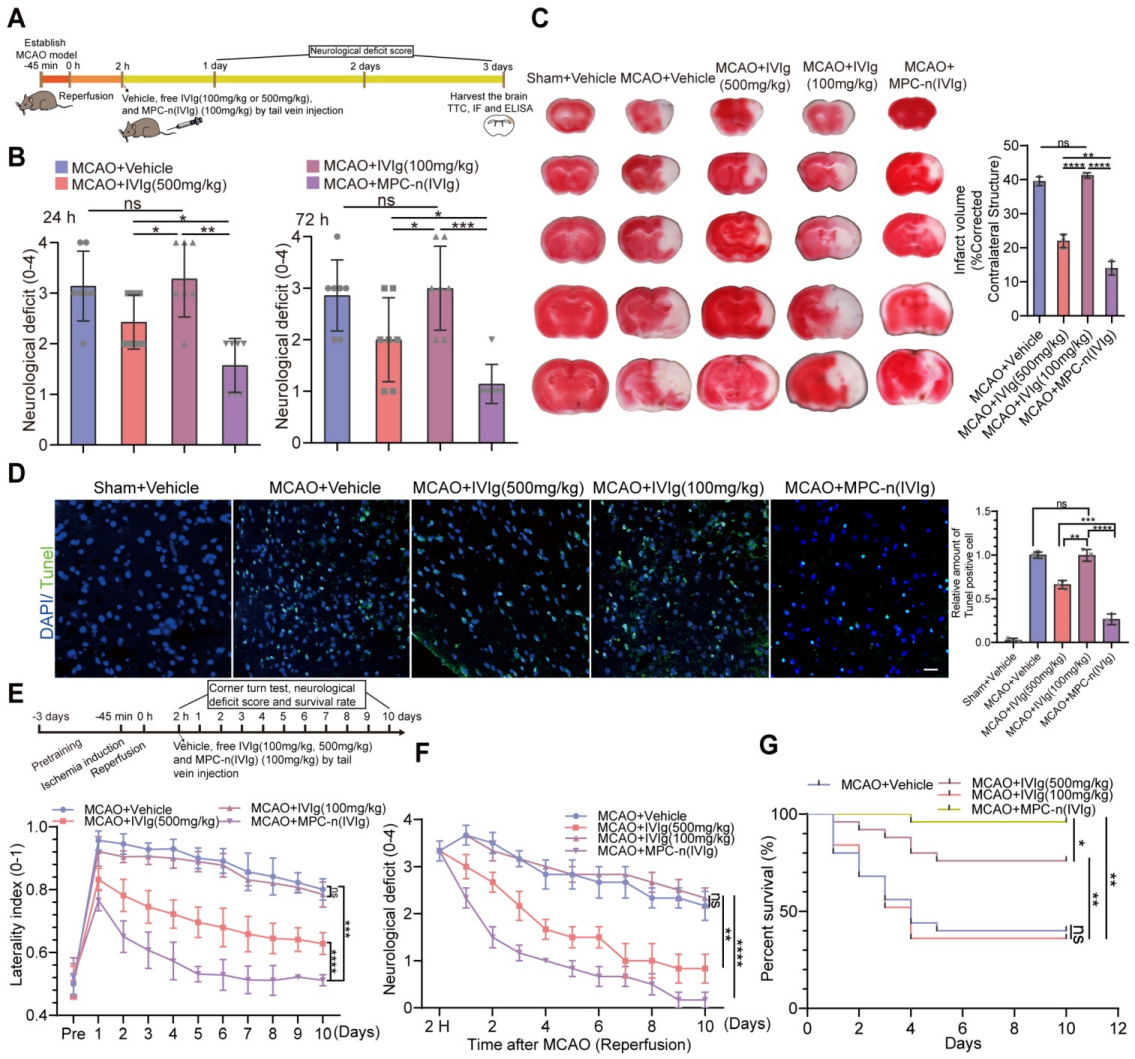
** Therapeutic efficacy of MPC-n(IVIg) in MCAO mice. A)** The experimental protocol used. **B)** Neurological deficit scores 24 or 72 h post-injection of vehicle (same volume), free IVIg (500 and 100 mg/kg), or MPC-n(IVIg) (100 mg/kg) 2 h post-ischemic-reperfusion in mice (n = 7). **C)** Representative images of TTC staining in the brain sections 3 d post-injection. The infarction area is indicated in the white color. The infarct volume was measured in the whole hemisphere and corrected by the contralateral structure (n = 3). MPC-n(IVIg) (100 mg/kg) was associated with a significant reduction in the infarct volume in the whole hemisphere compared to the vehicle and free IVIg (500 and 100 mg/kg) controls. **D)** Fluorescence of TUNEL staining in the ischemic penumbra 3 d post-injection of vehicle (same volume), free IVIg (500 and 100 mg/kg), or MPC-n(IVIg) (100 mg/kg) 2 h post-ischemic-reperfusion in mice. In TUNEL staining, normal and apoptotic cell nuclei stained blue and green, respectively. TUNEL (green), and DAPI (blue). The histogram quantifies the TUNEL assay (n = 3). Scale bar = 20 μm. **E)** The corner turn test was analyzed using the laterality index (number of right turns-number of left turns)/10 (n = 10). **F)** Daily neurological deficit score was collected over the 10 recovery days. Group 1: MCAO mice injected with vehicle (n = 5); group 2: MCAO mice injected with IVIg (500 mg/kg; n = 5); group 3: MCAO mice injected with IVIg (100 mg/kg; n = 5), or group 4: MCAO mice injected with MPC-n(IVIg) (100 mg/kg; n = 5) for 10 d after injection. **G)** Mouse survival rates 10 d post-injection of MPC-n(IVIg) (100 mg/kg) in the MCAO models (n = 25). Two hours after ischemic-reperfusion, the mice were administrated with vehicle, free IVIg (500 and 100 mg/kg), or MPC-n(IVIg) (100 mg/kg). All experiments were repeated independently three times. All data are presented as the mean ± S.D. ns, nonsignificant, *P < 0.05, **P < 0.01, or ***P < 0.001.

High dose of IVIg (≥500 mg/kg) is efficient in neuroprotection by regulating multiple critical biological processes during ischemic stroke 22, 23, 45. In our study, early administration (2 h) of MPC-n(IVIg) in the left hemisphere exhibited a greater than 5-fold accumulation compared to free IVIg in the MCAO models at 24 h post-injection. It is possible to reduce the effective dose of IVIg due to the targeting capability of MPC-n(IVIg) during ischemic stroke. To establish whether the early administration of low-dosage MPC-n(IVIg) could improve functional recovery, we used a therapeutic window paradigm (administered 2 h after reperfusion) and decreased the dosage of MPC-n(IVIg) to 100 mg/kg followed by recording the mouse neurological deficit scores. Furthermore, the brain tissues were harvested 3 d after treatment with several formulations and sections were stained with 2% triphenyl tetrazolium chloride (TTC) to quantify the infarcted brain tissue (Figure 5A). Low-dosage free IVIg (100 mg/kg) did not interrupt the evolution of the acute infarct volume and barely decreased the neurological scores by day three. MPC-n(IVIg) (100 mg/kg) treatment showed a reduction in the neurological deficits and infarct volume after 3 d (Figure 5B-C, Figure S12). Treatment with MPC-n(IVIg) (100 mg/kg) decreased the TUNEL-positive apoptotic cells (green) in the ischemic penumbra (Figure 5D), suggesting that the early administration of low-dosage MPC-n(IVIg) effectively reduced cell apoptosis in MCAO models. Subsequently, we demonstrated that administration of MPC-n(IVIg) (100 mg/kg) 2 h after reperfusion in MCAO models exerted therapeutic effects based on the corner turn test, and reduced neurological deficits and mortality for at least ten days (Figure 5E-G, Table S4). In addition, administration of a lower-dose MPC-n(IVIg) (50 mg/kg) did not decrease the neurological scores and barely improved survival by day ten (Figure S13). Our findings illustrated that during early ischemic-reperfusion, low-dosage MPC-n(IVIg) (100 mg/kg) effectively increased neuroprotective effects and reduced acute injury.

### Early administration of low-dosage MPC-n(IVIg) suppresses stroke-induced inflammation

**Figure 6:**
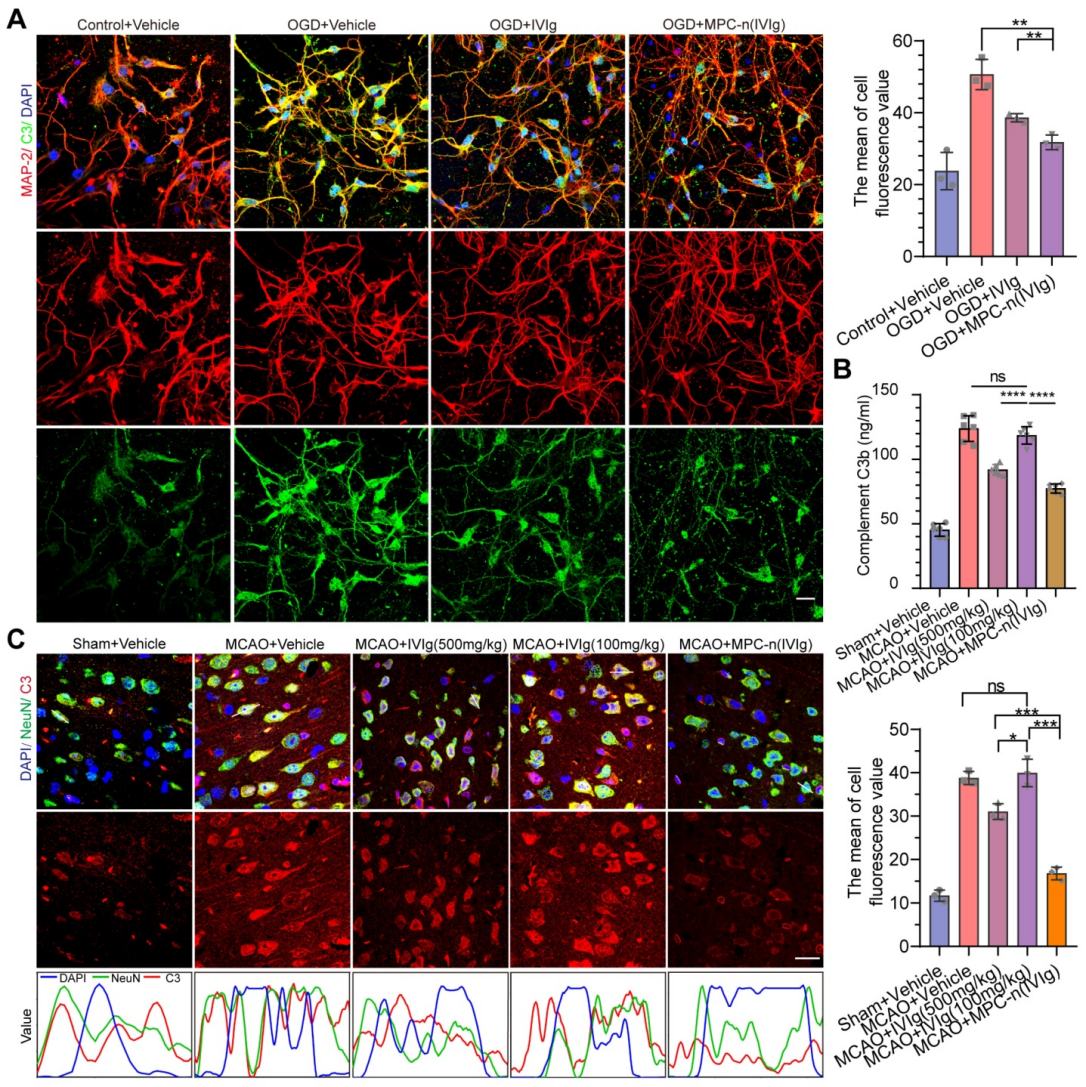
** Early administration of MPC-n(IVIg) inhibits the activation of neural C3 complement. A)** Immunofluorescence showed the expression of C3 in mature primary cortical neurons subjected to OGD for 12 h and incubated with vehicle, free IVIg, or MPC-n(IVIg) (10 mg/mL) for 24 h. Primary cortical neurons under normoxic conditions were the control. DAPI (blue), C3 (green), and MAP-2 (red). The histogram summarizes the relative fluorescence intensity of C3 in the primary cortical neurons (n = 3). Scale bar = 20 μm. **B, C)** MCAO mice were injected with vehicle, free IVIg (100 and 500 mg/kg), or MPC-n(IVIg) (100 mg/kg). **B)** ELISA was used to quantify C3b levels in brain homogenates from the ipsilateral hemisphere (left brain, n = 6). **C)** Immunofluorescence revealed the expression of C3 in ischemic penumbra (n = 3). Staining and counterstaining with nuclear marker DAPI (blue) indicated the colocalization of the neuronal nuclear marker NeuN (green) and C3 (red). Scale bar = 20 μm. The gray value is displayed in the line plots. All experiments were repeated independently three times. All data are presented as the mean ± S.D. ns, nonsignificant, *P < 0.05, **P < 0.01, or ***P < 0.001.

Directly following ischemic insult, neural cell death orchestrates inflammation characterized by the induction of the complement system, focal glial activation, and infiltration of peripheral immune cells, which contributes to the damage of the brain parenchyma and vasculature 4, 6, 9. High-dosage IVIg (500 mg/kg of body weight) treatment results in the suppression of stroke-induced increase of C3 and complement components 3b (C3b) levels during ischemic stroke 22. GO analysis of the cellular components and biological processes of the upregulated genes in the MCAO group showed that the upregulated genes were associated with complement and release of cytokines (Figure S14A-B). To verify the therapeutic efficacy of MPC-n(IVIg) in inhibiting complement in the double-coculture system, mature primary neurons and BV-2 cells were incubated with free IVIg and MPC-n(IVIg) for 24 h (Figure 6A, Figure S15), respectively. MPC-n(IVIg) inhibited C3 deposition in the pro-OGD-treated mature primary cortical neurons and BV-2 cells. Moreover, MCAO mice were injected with vehicle, free IVIg (500 and 100 mg/kg), or MPC-n(IVIg) (100 mg/kg) 2 h after reperfusion. After 3 d, we harvested the ipsilateral hemisphere and measured C3b levels using ELISA (Figure 6B). The MPC-n(IVIg)-treated group showed decreased levels of C3b compared to the free IVIg-treated group, indicating that administration of low-dosage MPC-n(IVIg) (100 mg/kg) was effective in reducing stroke-induced C3b during early ischemic-reperfusion. Immunofluorescence of the brain sections in the ischemic penumbra showed consistent data as of that obtained using ELISA (Figure 6C).

**Figure 7:**
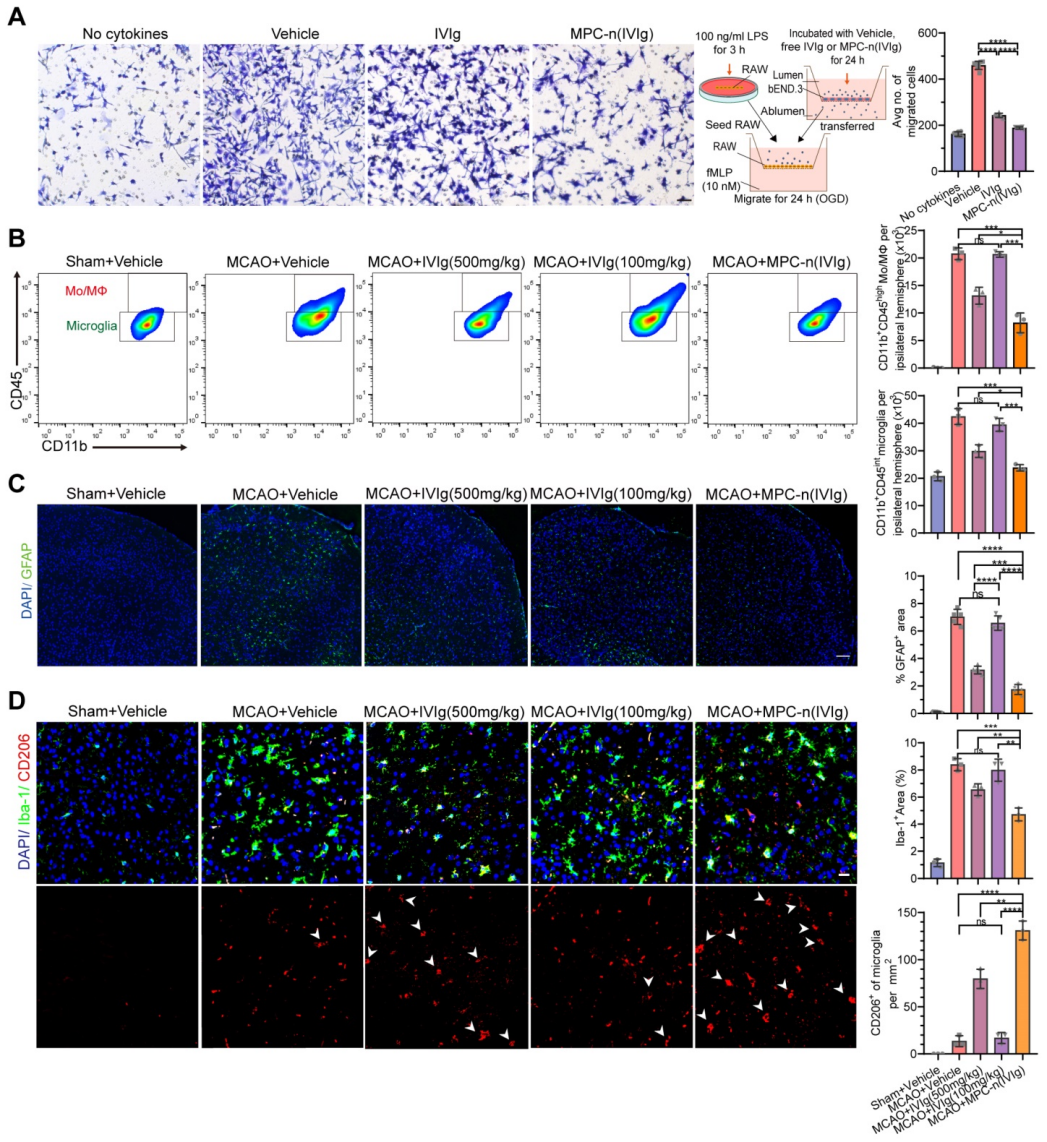
** Early administration of MPC-n(IVIg) after MCAO suppresses the infiltration of monocyte/macrophage, and activation of glial cells, and induces a protective phenotype in microglia. A)** Transwell migration assay of RAW 267.4 cells using 24-well plates with cell culture inserts. fMLP (10 nM) was added to the lower chamber (n = 7). RAW 267.4 cells activated by lipopolysaccharide (100 ng/mL) were incubated with vehicle, free IVIg, or MPC-n(IVIg) collected from the lower chamber of the BBB models and subjected to OGD for 24 h. Scale bar = 100 μm. **B)** Representative flow cytometry plots showing the two populations of CD45^+^ cells, infiltrating leukocytes (CD45 high), and microglia (CD45 intermediate) 3 d post-injection. **C)** Fluorescence images of astrocytes in the ischemic penumbra 3 d post-injection. GFAP (green), DAPI (blue). **D)** Fluorescence images of microglia (Iba-1) and M2 (CD206) in the ischemic penumbra 3 d post-injection. Iba-1 (green), CD206 (red), and DAPI (blue). Scale bar = 20 μm. All experiments were repeated independently three times. All data are presented as the mean ± S.D. ns, nonsignificant, *P < 0.05, **P < 0.01, or ***P < 0.001.

Furthermore, IVIg prevents the post-ischemic infiltration of monocytes/macrophages (Mo/MΦ) 45, suppresses glial cell activation 24, and induces a protective phenotype in microglia 46, 47. We studied the effect of MPC-n(IVIg) on the migration of macrophages using a transwell assay to mimic the migration of macrophages *in vivo* (Figure 7A). Control macrophages (absence of chemokine) showed low migration. In the other groups, we aliquoted N-formyl-Met-Leu-Phe (fMLP; 10 nM) in the lower chamber, and macrophages were seeded in the top chamber of the transwell. Macrophage migration was reduced after the treatment with MPC-n(IVIg) as compared to the migration of the free IVIg group. Moreover, the MCAO models were administered therapeutics 2 h after reperfusion. Brain tissues were obtained at 1, 2, and 3 d after treatment to analyse inflammation, respectively. We confirmed the dynamics of myeloid cell trafficking from the periphery to the ischemic brain using flow cytometry to assess macrophage recruitment to the ischemic areas following the administration of MPC-n(IVIg) (100 mg/kg, Figure 7B, Figure S16). CD11b^+^ myeloid cells were stained with anti-CD45 to distinguish CD45^int^-resident microglia from CD45^hi^ peripherally derived Mo/MΦ that were trafficked to the ischemic brain 48. The increase of infiltrating Mo/MΦ and resident microglia in the ipsilateral hemisphere was attenuated in the MCAO models treated with MPC-n(IVIg) (100 mg/kg) compared to those treated with vehicle or free IVIg (100 and 500 mg/kg). Moreover, we performed immunofluorescence to study the relative levels of GFAP, Iba-1, and CD206 (a marker of M2) in the ischemic penumbra with different treatments.

**Figure 8:**
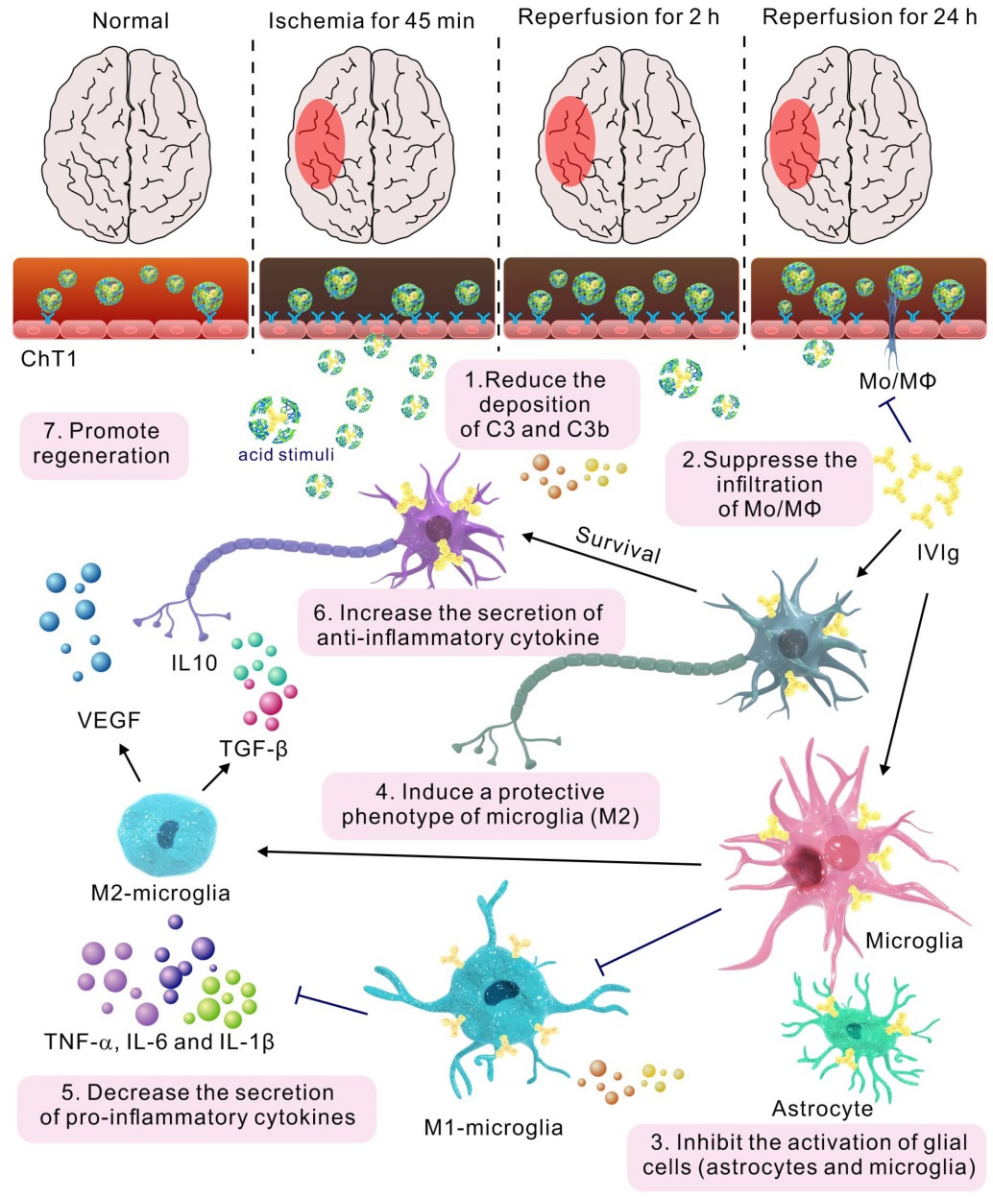
The mechanism of MPC-n(IVIg) modulating inflammatory response during ischemic stroke.

Microglial depletion has an adverse effect on reparatory mechanisms and post-stroke outcomes 46. MPC-n(IVIg) (100 mg/kg) did not completely inhibit microglial activation in the ipsilateral hemisphere after MCAO, and a higher relative abundance of Iba1^+^ cells was observed in the ipsilateral hemisphere compared to that in the contralateral hemisphere after MPC-n(IVIg) (100 mg/kg) treatment (Figure S17). MPC-n(IVIg)-treated (100 mg/kg) mice showed a lower density of GFAP^+^ astrocytes and Iba1^+^ microglia than those treated with free IVIg (100 and 500 mg/kg, Figure 7C-D). Activated microglia and infiltrating Mo/MΦ secrete pro-inflammatory cytokines, including TNF-α, interleukin-6 (IL-6), and interleukin-1 (IL-1β), which play a central role in neuroinflammation and lead to brain damage during ischemic stroke 49, 50. ELISA showed that MPC-n(IVIg) (100 mg/kg) decreased these cytokines in the ipsilateral hemisphere (Figure S18A-C) as compared to free IVIg (100 and 500 mg/kg). Moreover, MPC-n(IVIg) (100 mg/kg) increased the abundance of CD206^+^ microglia cells in the ischemic penumbra as compared to that using free IVIg (100 and 500 mg/kg; Figure 7D). M2 microglia cells release transforming growth factor β (TGF-β) and interleukin-10 (IL-10) to resist inflammation and vascular endothelial growth factor (VEGF) to promote regeneration 49. Our findings revealed that MPC-n(IVIg) (100 mg/kg) increased the levels of TGF-β, IL-10, and VEGF in the brain homogenate of the ipsilateral hemisphere as compared to free IVIg (100 and 500 mg/kg; Figure S18D-F)**.** Our results indicated that the early administration of low-dosage MPC-n(IVIg) (100 mg/kg) alleviated the inflammatory response to enhance neuroprotection by inhibiting the deposition of C3 and C3b, suppressing the infiltration of Mo/MΦ, activating of glial cells (astrocytes and microglia), and inducing a protective phenotype in microglia (Figure 8).

## Discussion

Inflammation is important in the initiation and progression of ischemic stroke 51. Immunomodulatory therapies are an appropriate supplement after reperfusion therapy, such as thrombolysis or thrombectomy, to prevent brain damage and neurological deficits during ischemic stroke 5, 51, 52. Several strategies have been explored to deliver therapeutics across the BBB, such as therapeutic fusion proteins conjugated with ligand or peptide (e.g., transferrin receptor monoclonal antibody) via receptor-mediated transcytosis 53, 54. Although these delivery systems target the BBB-specific receptor and enhance BBB penetration, the delivery of therapeutics to the CNS is still suboptimal due to undesired biodistribution, which may cause unwanted toxicities, and further insufficient enrichment in the target tissues 55-57. Besides, the fusion proteins conjugated with ligand or peptide usually face the problem of stability *in vivo* 57. Moreover, the infusion of therapeutics has also been employed 35. This is unsuccessful due to difficulties in modulating the release kinetics of therapeutics and high risks associated with invasive surgeries. Furthermore, the delivery of therapeutics to the CNS by penetrating the BBB with osmotic disruption (e.g., mannitol) has been investigated 35, 58. However, the dose and administration schedule of compounds needs to be well optimized, and its efficacy remains to be validated. MPC-nanocapsules were formed by encapsulating the protein within a polymer shell that had PMPC as the main component. PMPC endows the nanocapsules with excellent performances, including reducing protein absorption, prolonging circulation time, and improving biocompatibilities 59-61. Despite decades of efforts, patients with ischemic stroke cannot benefit from immunomodulatory therapy due to the presence of the BBB and the high accumulation in off-target organs or poor accumulation in target ischemic region, respectively 11, 15, 62. Here, we described a novel therapeutic regimen including the early administration of MPC-n(IVIg) in promoting BBB penetration and selectively targeting ischemic areas to deliver IVIg in the ischemic region, thereby alleviating neurological damage during ischemic stroke by modulating stroke-induced inflammation.

IVIg was encapsulated using in situ polymerization with MPC monomers and EGDMA crosslinking. MPC-nanocapsules did not affect the cell viability, BBB integrity, and *in vitro* or *in vivo* inflammatory response owing to their excellent biocompatibility. MPC-nanocapsules cross the BBB via ChT1-mediated transcytosis 28, 29. We demonstrated that the overexpression of endothelial-cell ChT1 was dependent on HIF-1α during ischemia and gradually decreased with reperfusion. Moreover, MPC-nanocapsules effectively enhanced BBB penetration and reduced the off-target effect through selective accumulation in ischemic regions due to the overexpression of ChT1 in endothelia cells during ischemic stroke. Immunomodulatory therapies have a wide therapeutic window and are efficacious when administered up to 12-24 hours after stroke; early treatment leads to better outcomes 6. There was greater IVIg accumulation in the ischemic brain when administered after 2 h of reperfusion than after 24 h. Therefore, MPC-n(IVIg) should be promptly administered. Therapeutic preparations of high-dose IVIg are efficacious anti-inflammatory and immunomodulatory treatments for a growing number of neurological diseases, such as multifocal motor neuropathy, neuromyelitis optica, Alzheimer's disease, and ischemic stroke 19, 22, 63. Moreover, the administration of high-dose IVIg (500 mg/kg of body weight) 1 h after MCAO decreases ischemic stroke-induced neurological impairment 3 d after reperfusion 22. However, adverse reactions occur more frequently in patients administered with high-dose IVIg compared to low-dose administered patients 19. Early administration (2 h) of MPC-n(IVIg) reduced brain infarction and neurological deficits after reperfusion with a 5-fold lower dose (100 mg/kg). Moreover, the early administration of low-dosage MPC-n(IVIg) improved therapeutic outcomes by modulating inflammation. This was attributed to the reduced deposition of C3, suppressed infiltration of macrophages, glial cells activation, and induction of a protective microglial phenotype. However, counteraction of the inflammatory response to ischemic insults may ameliorate tissue damage in the acute phase but compromise repair mechanisms and worsen long-term outcomes 5, 64. In this study, transient and ischemia-specific inflammatory modulation by MPC-n(IVIg) did not adversely affect reparatory processes.

Despite the encouraging selective accumulation and efficacy of MPC-n(IVIg) in MCAO mice, there are several limitations in this study before clinical translation. We have not specifically addressed whether there is other transcytosis pathway for promoting BBB penetration and selective accumulation in ischemic regions. In addition, the overexpression of ChT1 in a HIF-1α-dependent manner is limited to young MCAO mice, and it may not completely recapitulate or summarize in patients with ischemic stroke in clinical trials. Thus, in accordance with the Stroke Treatment Academic Industry Roundtable recommendations 65, there is a need for additional confirmatory researches in different ischemia times, gender, aged (10-month-old) mice, and various preclinical models such as nonhuman primates to further validate this delivery system for potential translation and some differences could occur when these results are translated into clinical trials. Moreover, we only evaluated the therapeutic effect of MPC-n(IVIg) in the context of ischemic stroke. Whether such an approach would be effective in other CNS disorders remains to be determined.

In summary, we have developed a feasible approach to enable the efficient and selective delivery of therapeutic IVIg to ischemic regions after ischemic stroke. Encapsulation of IVIg within the MPC-nanocapsules facilitated BBB penetration and selectively targeted to the ischemic region result from the overexpression of endothelial-cell ChT1 in the ischemic areas. Moreover, the early administration of low-dosage MPC-n(IVIg) decreased neurological deficits and improved therapeutic outcomes by modulating inflammation. This study sheds light on the mechanism underlying effective BBB penetration and selective accumulation of MPC-n(IVIg) in the ischemic region during ischemic stroke and its potential feasibility in clinical application.

## Supplementary Material

Supplementary figures and tables.
