# Supplementary material for: Early administration of MPC-n(IVIg) selectively accumulates in ischemic areas to protect inflammation-induced brain damage from ischemic stroke
Source: Theranostics. 2021 Jul 13;11(17):8197–217. doi: 10.7150/thno.58947 (PMC8344004; doi:10.7150/thno.58947)
Supplement: Supplementary file 1 — Supplementary figures and tables. [file thnov11p8197s1.pdf]

Supporting Information

Early administration of MPC-n(IVIg) selectively accumulates in ischemic areas to protect inflammation-induced brain damage from ischemic stroke

Weili Jin[1], Ye Wu[1], Ning Chen[2], Qixue Wang[1], Yunfei Wang[1], Yansheng Li[1], Sidi Li[2], Xing Han[2], Eryan Yang[1], Fei Tong[1], Jialing Wu[3*], Xubo Yuan[2*], and Chunsheng Kang[1*]

Affiliations:

1. Department of Neurosurgery, Tianjin Medical University General Hospital, Laboratory of Neuro-oncology, Tianjin Neurological Institute, Key Laboratory of Post-Neurotrauma Neuro-Repair and Regeneration in Central Nervous System, Ministry of Education and Tianjin City, Tianjin 300052, China.

2. Tianjin Key Laboratory of Composite and Functional Materials, School of Materials Science and Engineering, Tianjin University, Tianjin 300072, China.

3. Tianjin Key Laboratory of Cerebral Vascular and Neurodegenerative Diseases, Tianjin Neurosurgical Institute, Tianjin Huanhu Hospital, Tianjin 300350, China. Department of Neurology, Tianjin Huanhu Hospital, Tianjin 300350, China.

*Correspondence: Jialing Wu (wywjl2009@hotmail.com), Xubo Yuan (xbyuan@tju.edu.cn), Chunsheng Kang (kang97061@tmu.edu.cn)

These authors contributed equally: Weili Jin, Ye Wu

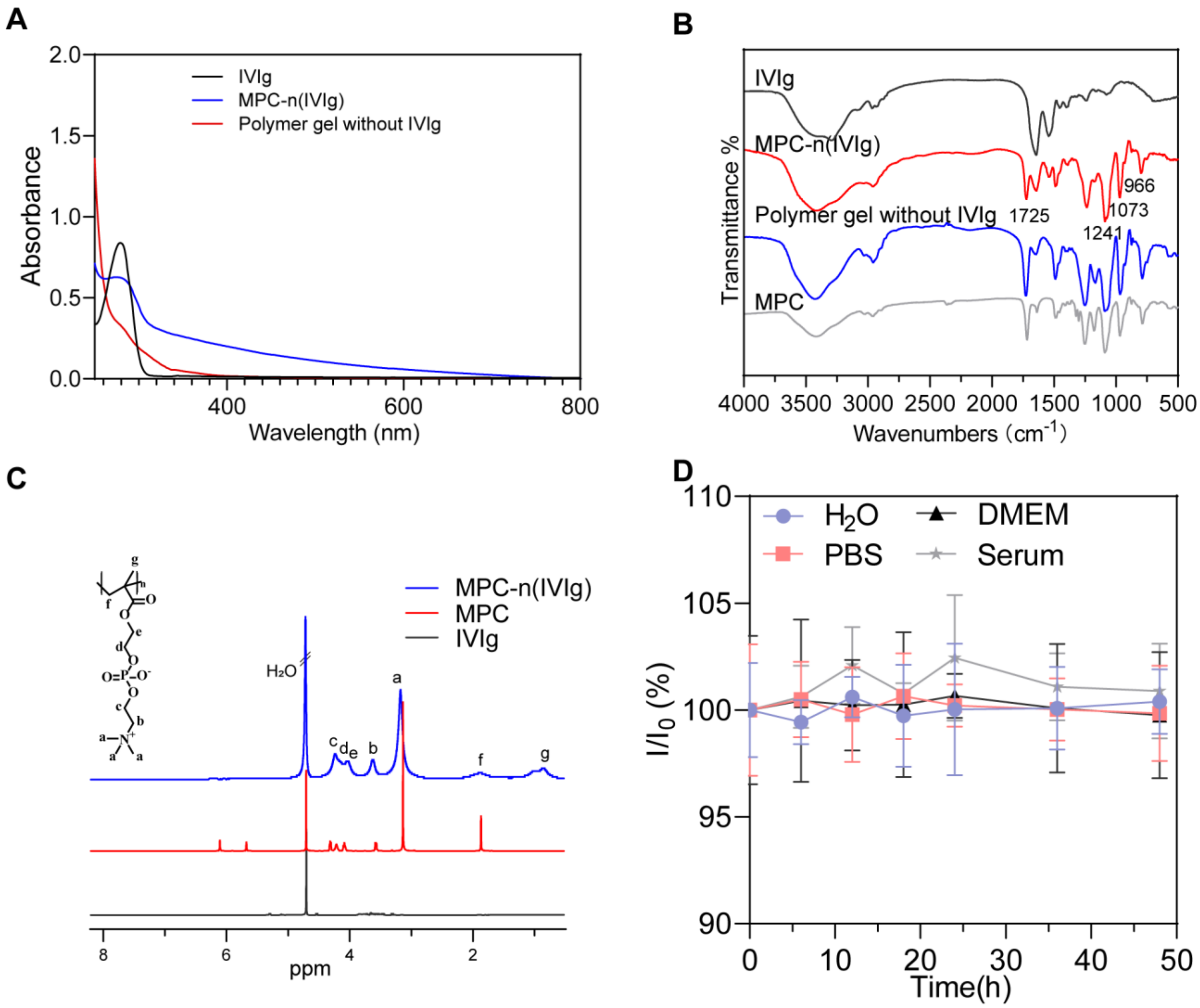

Figure S1. Characteristics of MPC-n(IVIg).

A) UV-vis spectra of MPC-n(IVIg).

B) FT-IR spectra of MPC, free IVIg, polymer gels without IVIg, and MPC-n(IVIg) after lyophilization.

C) ^{1}H NMR spectra of MPC, free IVIg, and MPC-n(IVIg) recorded in D_2O at a concentration of 5 mg/mL.

D) The stability of MPC-n(IVIg) in H_2O, PBS, DMEM, and serum at 37°C, determined by monitoring particle size (diameter) for 48 h (n = 3). All experiments were repeated independently three times. All data are presented as the mean ± S.D. ns, nonsignificant.

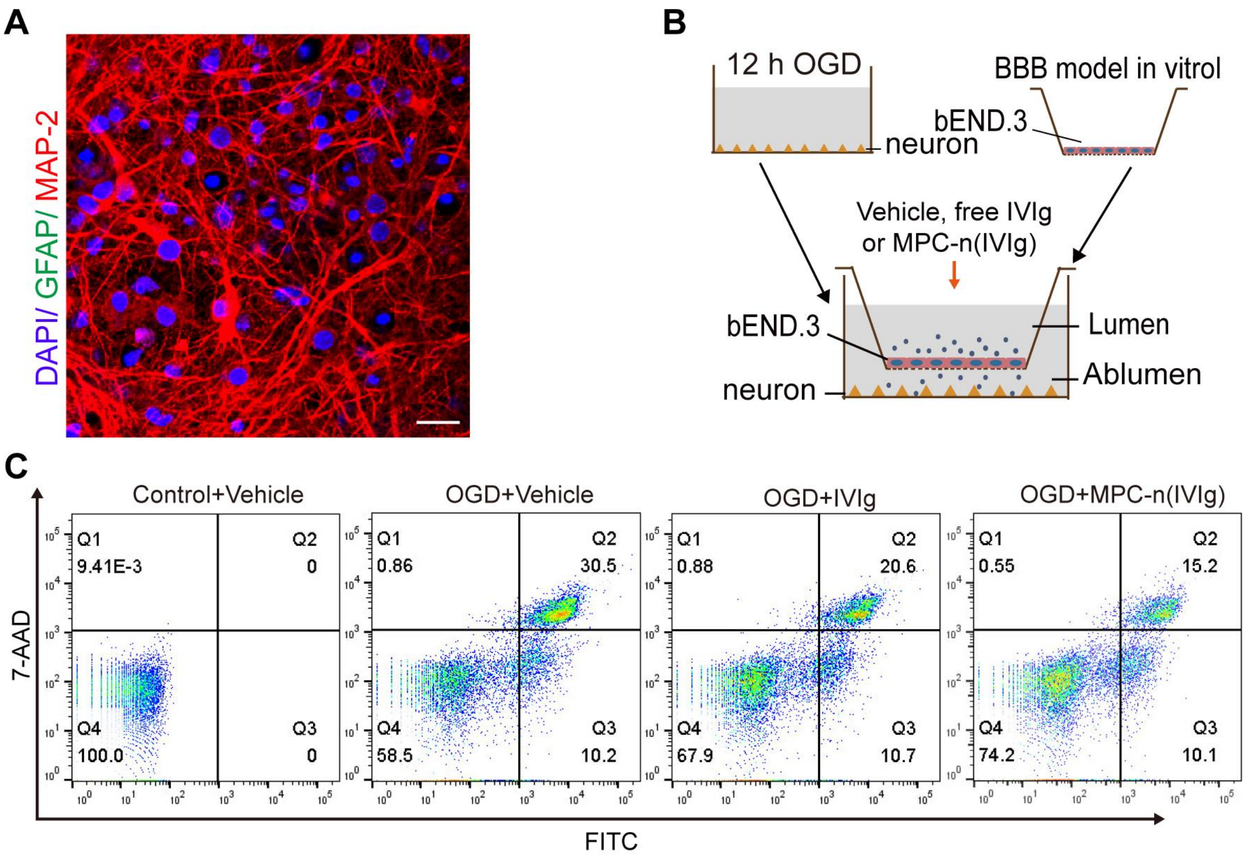

Figure S2. Activity of IVIg released from MPC-n(IVIg) in mature primary cortical neurons.

A) Fluorescence imaging of the mature primary mouse neurons (15 d *in vitro*). DAPI, mouse anti-MAP-2 antibody, and rabbit anti-GFAP antibody were used to stain the nuclei, mature neurons, and astrocytes, respectively. Scale bar = 50 μm.

B) Schematic illustration of the transwell assay with endothelial cells in double-coculture with mature primary cortical neurons.

C) Representative flow cytometry showing the effect of vehicle, free IVIg, and MPC-n(IVIg) (10 mg/mL) on the apoptosis of mature primary cortical neurons. In each panel, the lower left quadrant (Q4) shows negative for both 7AAD and Annexin V-FITC cells, which indicate viable cells, while the upper left quadrant (Q1) shows only 7AAD cells, which are necrotic. The upper right quadrant (Q2) shows both Annexin and 7AAD positive cells, which are in the late apoptosis,

while the lower quadrant right (Q3) shows Annexin-positive cells, which are in the early apoptosis. All experiments were repeated independently three times.

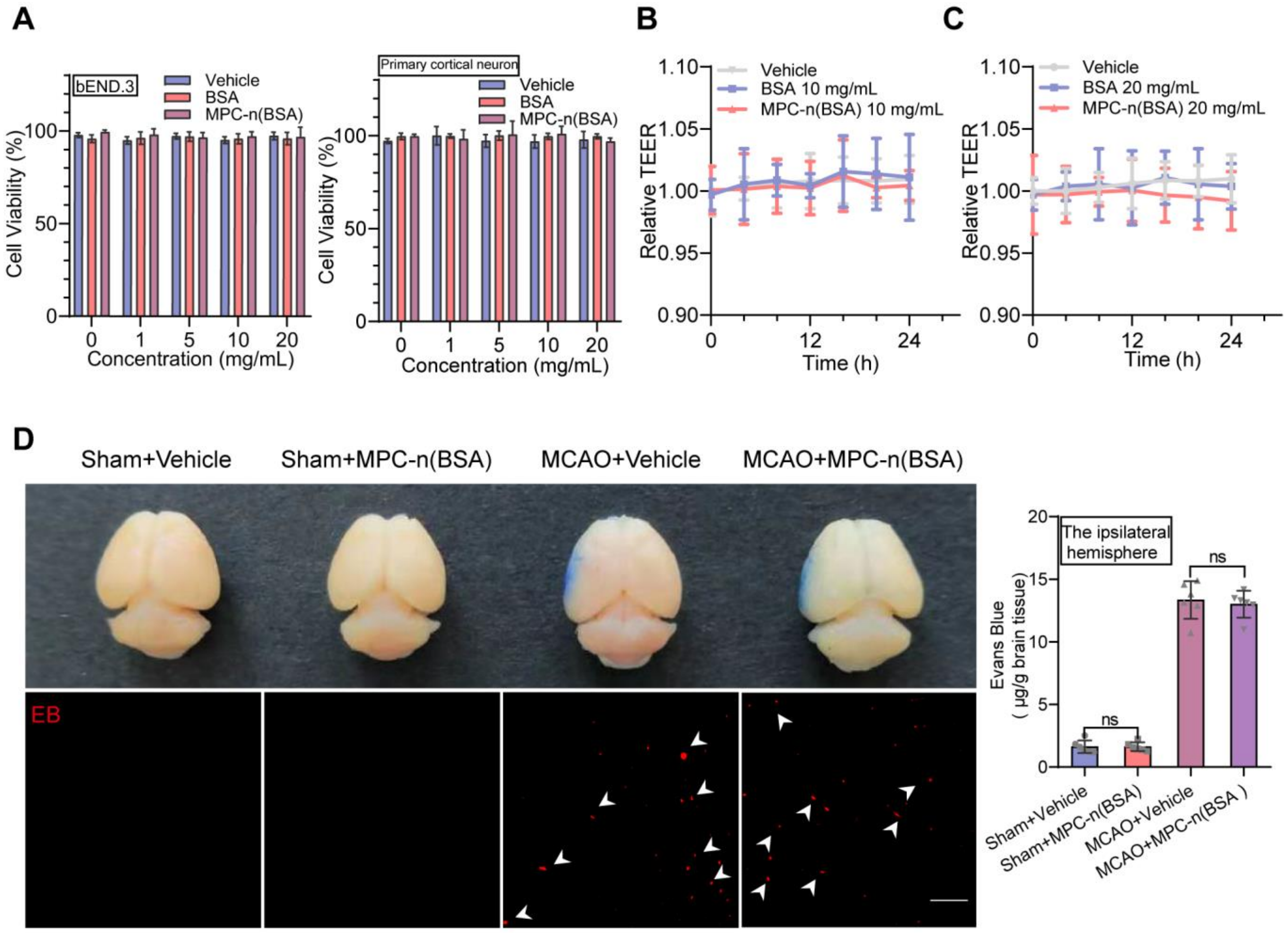

Figure S3. MPC-nanocapsules do not affect cell viability and BBB integrity *in vitro* and *in vivo*.

A) Cell viability assays after incubating bEND.3 cells and primary cortical neurons with different concentrations of vehicle, BSA, or MPC-n(BSA) for 24 h (n = 6).

B, C) Long-term impact of different concentrations of MPC-n(BSA) on cell integrity for 24 h using Electrical Cell Impedance Sensing array (n = 6). Transendothelial electrical resistance

from the vehicle group measurement was set to one. Each measurement was expressed as a relative value.

D) Evans blue (EB) dye extravasation analysis of sham and MCAO mice treated with vehicle or MPC-n(BSA) 100 mg/kg for 24 h to evaluate BBB integrity. Upper: digital photographs of brains stained by permeating EB. Lower: corresponding representative immunofluorescence of the brain sections showing red fluorescence of EB under excitation at a wavelength of 620 nm. Scale bar = 50 μm. Right: EB extravasation of brain homogenates from the ipsilateral hemisphere (left brain). Data are expressed as μg of EB per g of brain tissue (n = 6). All experiments were repeated independently three times. All data are presented as the mean ± S.D. ns, nonsignificant.

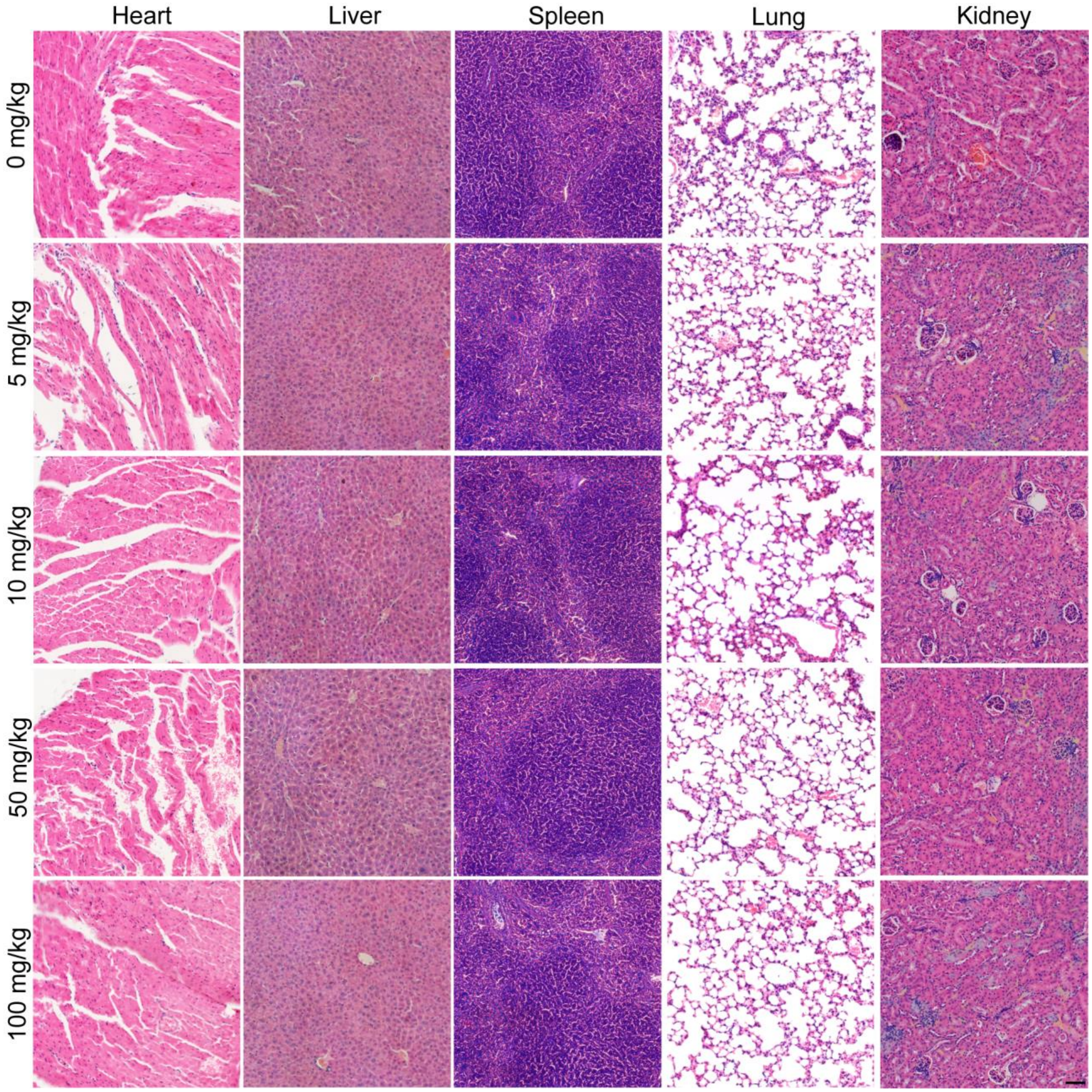

Figure S4. MPC-nanocapsules are non-toxic *in vitro* in the long term.

H&E-stained tissue sections (heart, liver, spleen, lung, and kidney) from mice after one intravenous injection of MPC-n(BSA) at different doses (0, 5, 10, 50, and 100 mg/kg) for 30 d (scale bar = 50 μm).

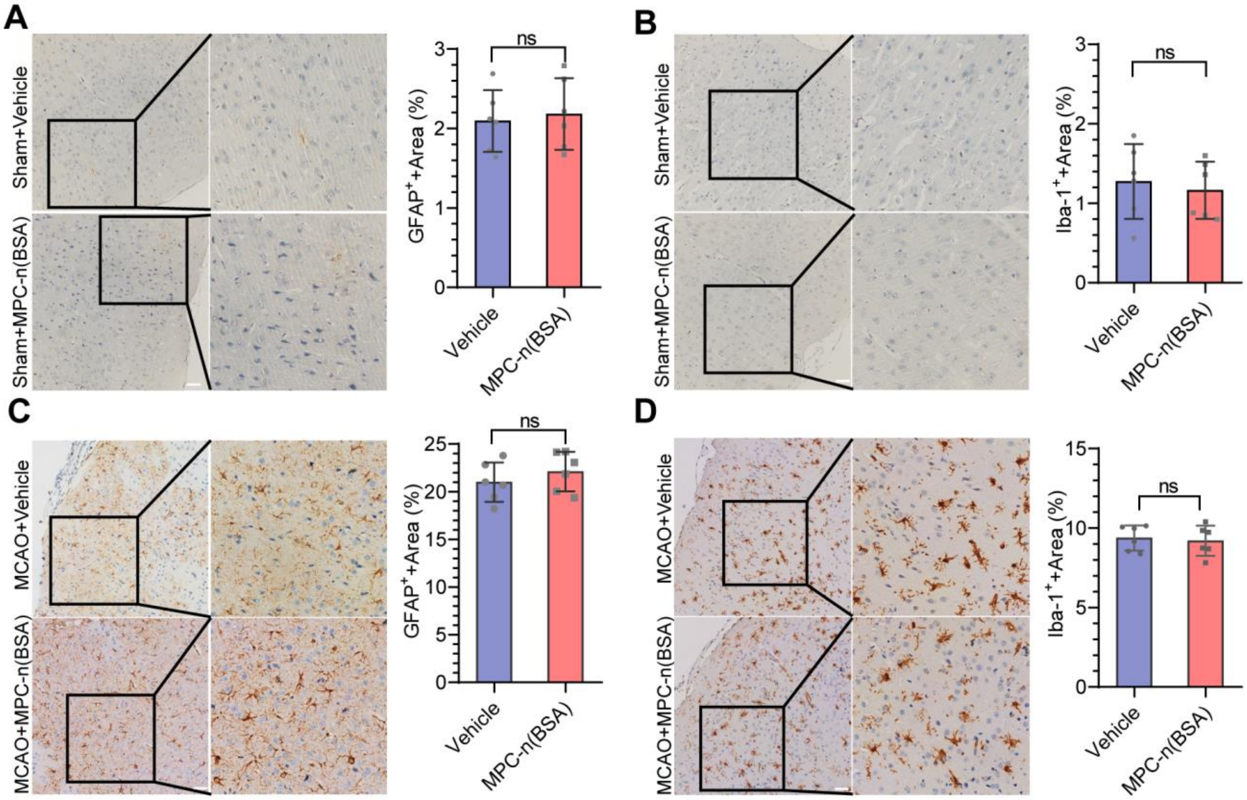

Figure S5. Systemic administration of MPC-nanocapsules does not activate microglia and astrocytes *in vivo*.

A-D) Immunohistochemistry images and quantification of astrocytes and microglia in the brains of the sham or MCAO models treated with vehicle and MPC-n(BSA) (100 mg/kg), respectively. **A, C)** Immunohistochemistry of the mouse brain stained with primary antibody to GFAP (n = 6). Quantification of the GFAP-positive area in the brains of mice treated with vehicle or MPC-n(BSA) using NIH ImageJ. **B, D)** Immunohistochemistry of the mouse brain stained with primary antibody to Iba-1 (n = 6). Quantification of the Iba-1-positive area in the brains of mice treated with vehicle or MPC-n(BSA) using NIH ImageJ. Scale bar = 50 μm. All experiments were repeated independently three times. All data are presented as the mean ± S.D. ns, nonsignificant.

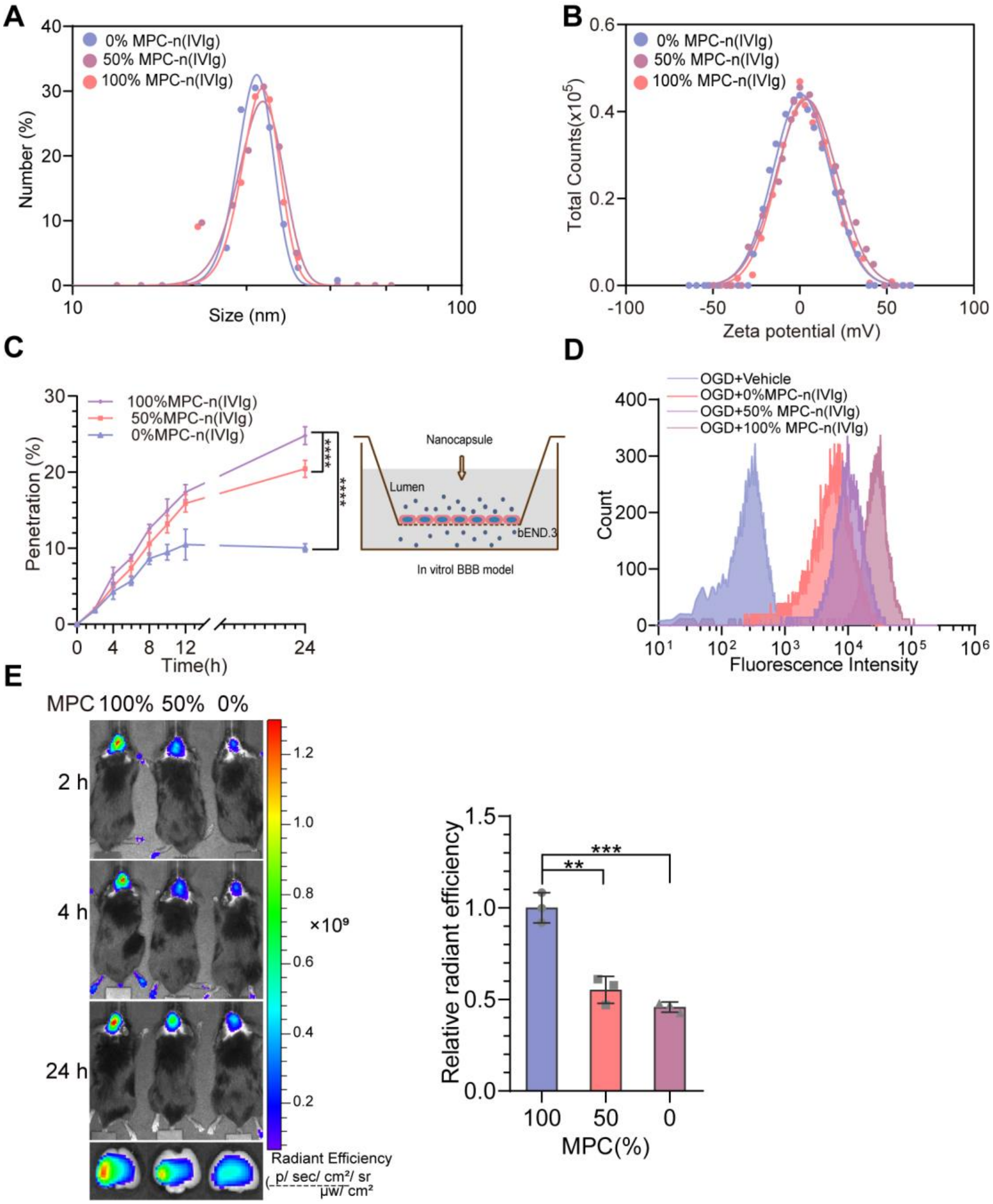

Figure S6. MPC as the targeting ligand on MPC-nanocapsules.

A) Size distribution of series nanocapsules with different MPC-moiety contents measured by dynamic light scattering.

B) Zeta potentials of series nanocapsules with different MPC-moiety contents.

C) Quantification of series nanocapsules with different contents of the MPC (100%, 50%, and 0%) penetration through the endothelial cells (bEND.3) in the transwell migration assay across

different time points in the groups: 100%, 50%, and 0% MPC-n(IVIg) (1 mg/mL, n = 3). Schematics of the bEND.3 transwell assay for constructing the *in vitro* BBB models.

D) Fluorescence-activated cell sorting of bEND.3 cells subjected to 12 h pro-OGD in a 24-well plate incubated with Cy5-labeled nanocapsules (1 mg/mL) with different contents of MPC for 6 h under normoxic conditions.

E) *In vivo* images of the MCAO mice at 2, 4, and 24 h after a one-time injection of 10 mg/kg MPC-n(IVIg) (Cy5-labeled IVIg) made with different MPC contents, and *ex vivo* fluorescence imaging of the isolated brains 24 h after injection (n = 3). The histogram summarizes the relative radiant efficiency of the isolated brain tissue. All experiments were repeated independently three times. All data are presented as the mean ± S.D. ns, nonsignificant, *P < 0.05, **P < 0.01, or ***P < 0.001.

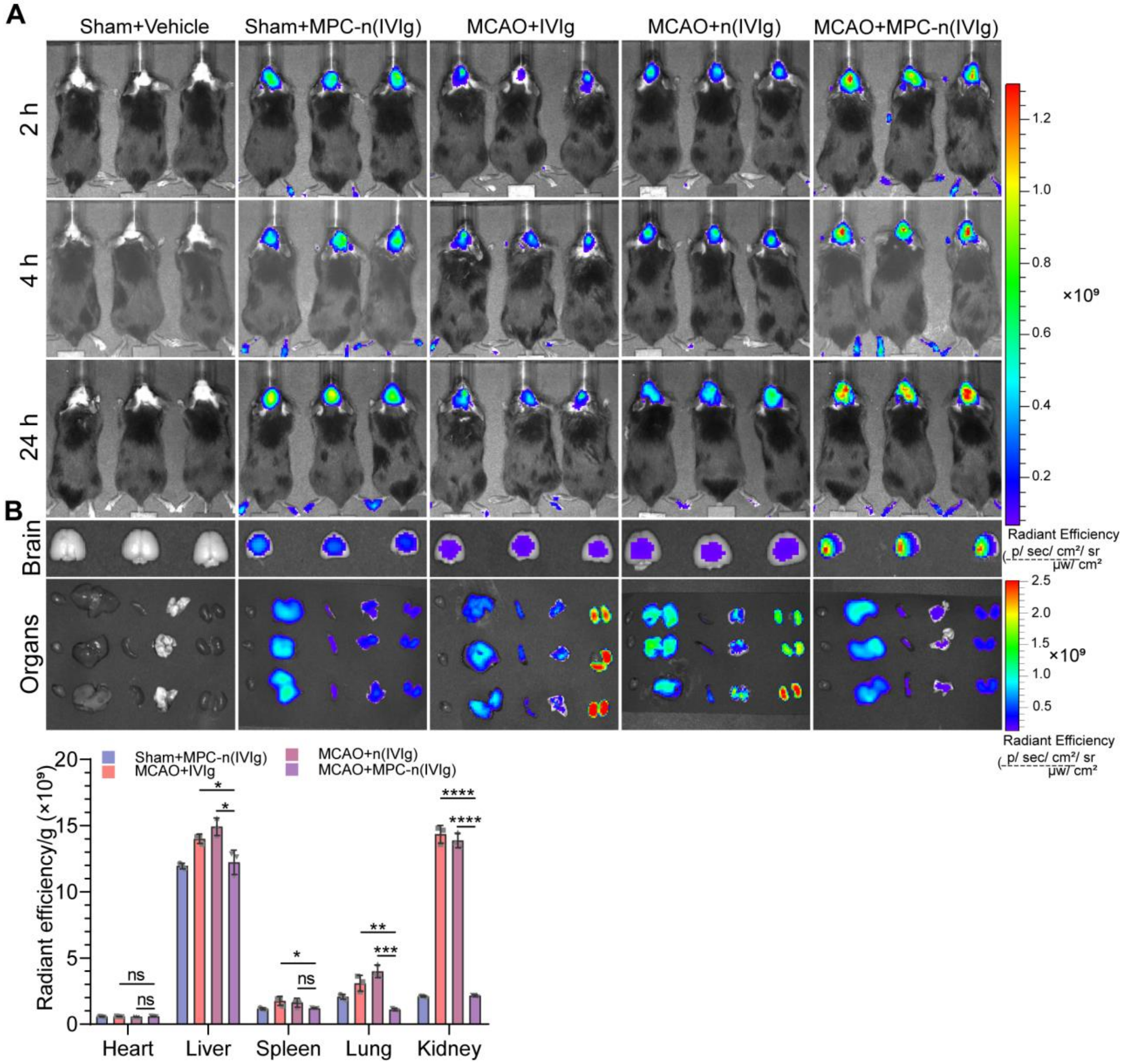

Figure S7. Whole-body and organ biodistribution of nanocapsules.

A, B) *In vivo* imaging of Cy5 signal 2, 4, and 24 h after intravenous injection of vehicle, free IVIg, n(IVIg), or MPC-n(IVIg) (10 mg/kg) in the MCAO or sham models. Isolated tissues of the treated mice were harvested 24 h post-injection. Quantification of IVIg accumulated in the different organs 24 h after the different treatments (n = 3 biologically independent samples). All experiments were repeated independently three times. All data are presented as the mean ± S.D. ns, nonsignificant, *P < 0.05, **P < 0.01, or ***P < 0.001.

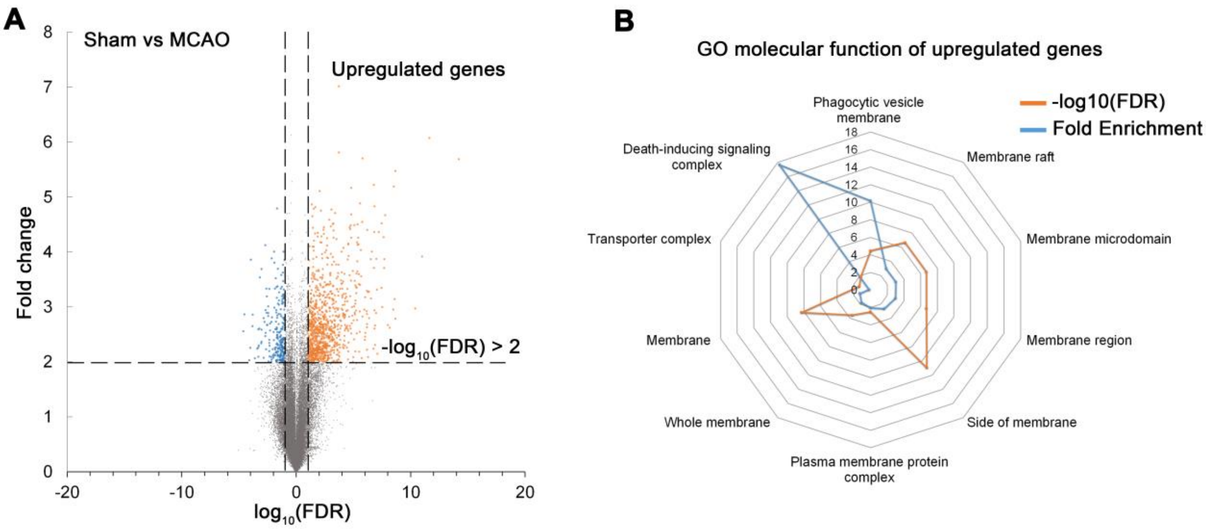

Figure S8. Membrane transporter is involved in ischemic stroke.

A) The upregulated genes (-$\log_{10}$(FDR)>2, fold change>1) between the sham and MCAO groups cells were shown in the volcano plot.

B) Gene ontology (GO) cellular component of the upregulated genes in the MCAO group was involved in the membrane, transporter complex, and death-inducing signaling complex.

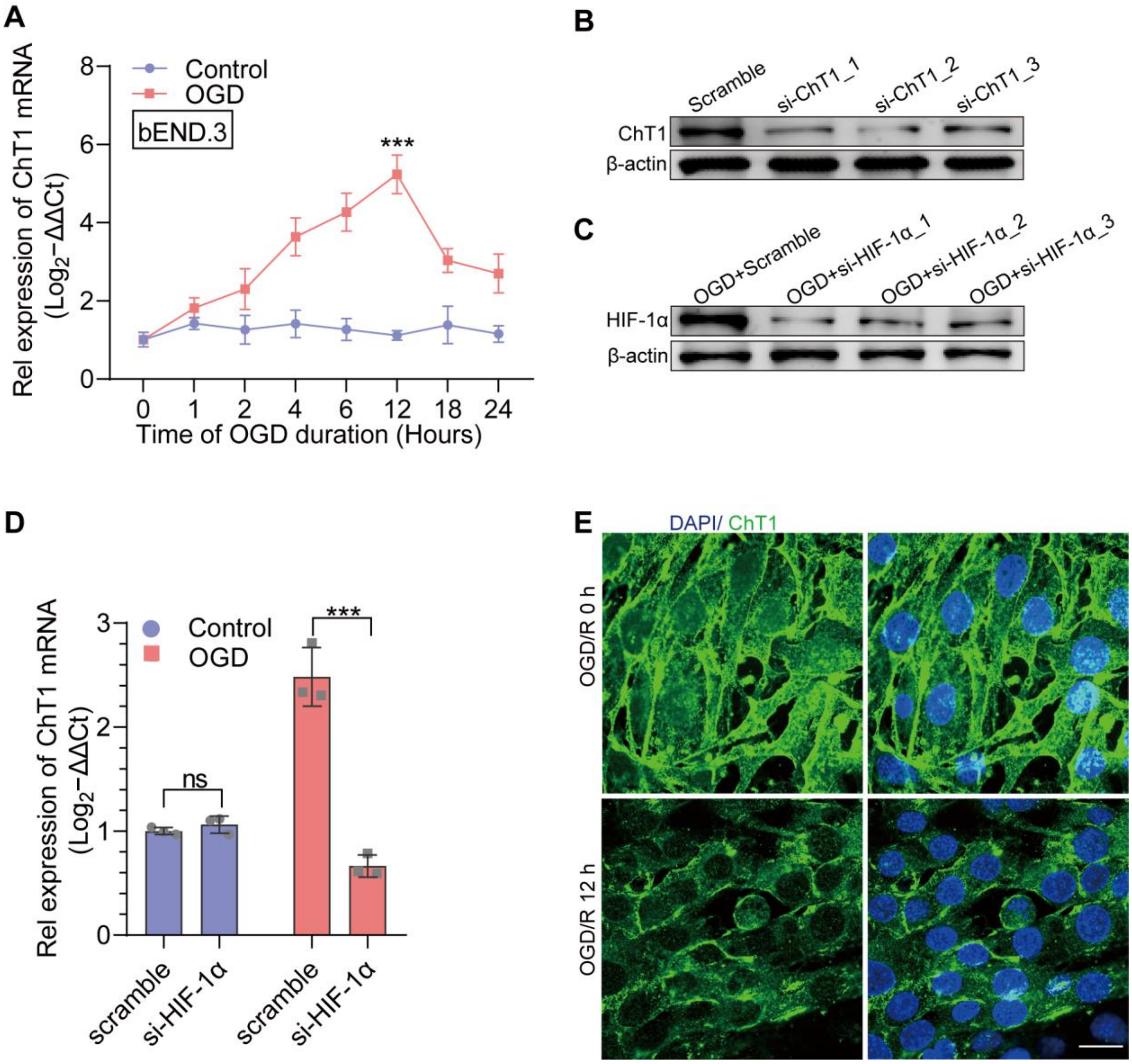

Figure S9. Overexpression of ChT1 depends on HIF-1α in endothelial cells after OGD.

A) bEND.3 cells were subjected to OGD for 1, 2, 4, 6, 12, 18, and 24 h and ChT1 mRNA expression was determined using qRT-PCR (n = 3).

B) Representative Western blot analysis of ChT1 in bEND.3 cells infected with siRNAs for 48 h.

C) Representative Western blot analysis of HIF-1α in bEND.3 cells infected with siRNAs for 48 h and subsequently subjected to OGD for 12 h.

D) bEND.3 cells were treated with siHIF-1α and subjected to OGD for 12 h, followed by determination of ChT1 mRNA expression using qRT-PCR (n = 3).

E) Immunofluorescence revealed the expression of ChT1 in bEND.3 cells after being subjected to OGD for 12 h with or without reperfusion for 12 h. ChT1 (green) and DAPI (blue). Scale bar = 50 μm. All experiments were repeated independently three times. All data are presented as the mean ± S.D. ns, nonsignificant, *P < 0.05, **P < 0.01, or ***P < 0.001.

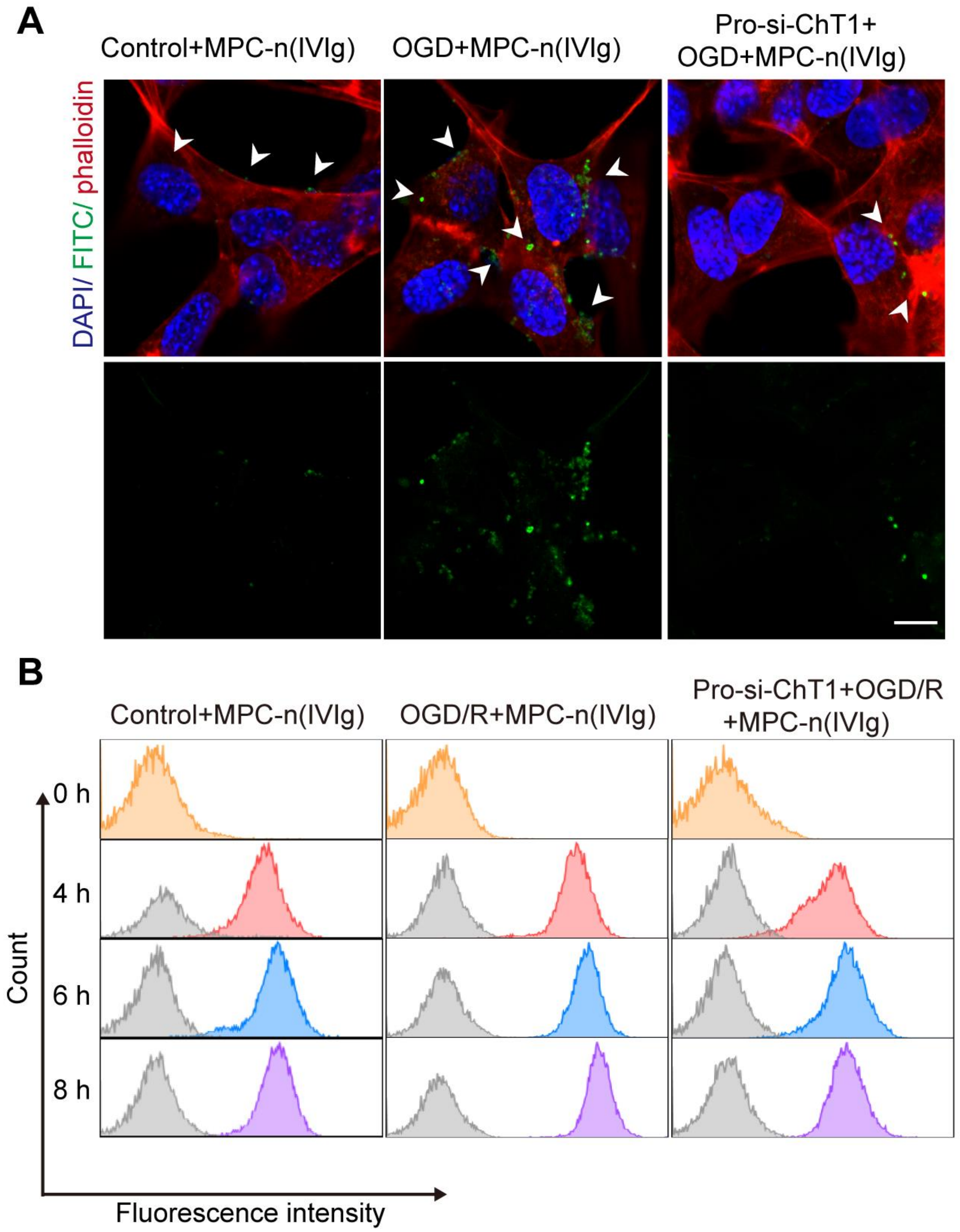

Figure S10. Uptake of MPC-n(IVIg) in bEND.3 cells after reoxygenation.

A) Fluorescent images of bEND.3 cells following incubation with MPC-n(IVIg) for 4 h with reperfusion in the control, OGD/R, and pro-siChT1+OGD/R groups. Scale bar = 20 μm.

B) Flow cytometry revealed the uptake of MPC-n(IVIg) by bEND.3 cells in the control, OGD, and pro-siChT1+OGD groups. All experiments were repeated independently three times. ns, nonsignificant, $^{*}P < 0.05$, $^{**}P < 0.01$, or $^{***}P < 0.001$.

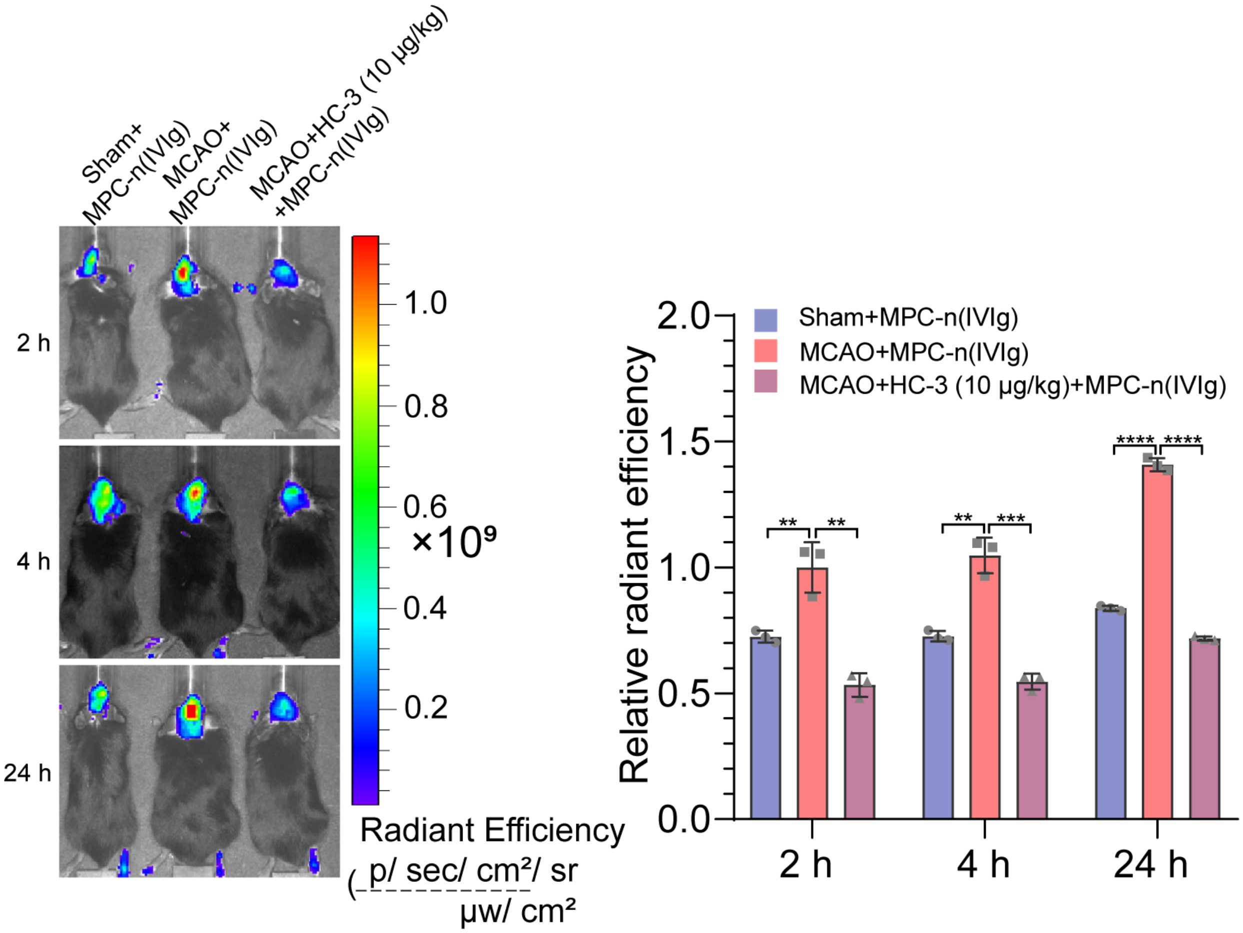

Figure S11. Inhibition of ChT1 represses accumulation of MPC-n(IVIg) in brains during ischemic stroke.

In vivo images of treated mice 2, 4, and 24 h after intravenous injection of MPC-n(IVIg) (10 mg/kg) in the sham and MCAO models with or without intraperitoneally preinjected with HC-3 (10 μg/kg) 20 mins prior to the injection of MPC-n(IVIg). The histogram summarizes the relative radiant efficiency of the brain tissue (n = 3). All experiments were repeated independently three times. All data are presented as the mean ± S.D. ns, nonsignificant, $*P < 0.05$, $**P < 0.01$, or $***P < 0.001$.

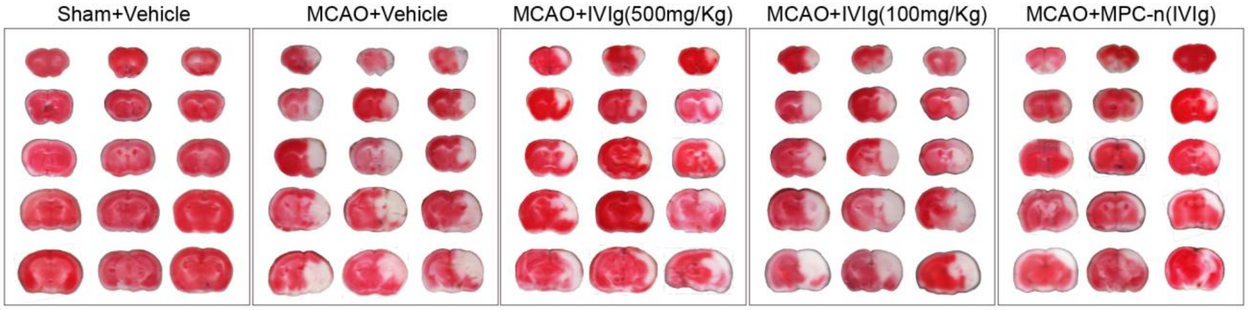

Figure S12. All TTC staining images in the brain sections 3 d post-injection (n = 3).

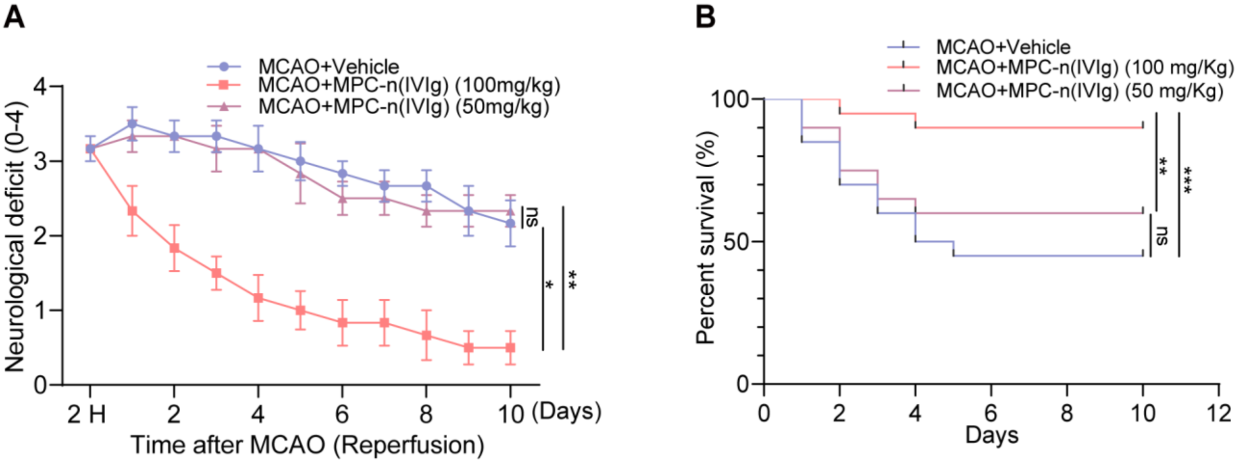

Figure S13. Therapeutic efficacy of MPC-n(IVIg) at two different doses in MCAO mice.

A) Daily neurological deficit score was collected over the 10 recovery days. Group 1: MCAO mice injected with vehicle (n = 6); group 2: MCAO mice injected with IVIg (100 mg/kg; n = 6); or group 3: MCAO mice injected with IVIg (50 mg/kg; n = 6) for 10 d after injection.

B) Mouse survival rates 10 d post-injection of MPC-n(IVIg) (100 mg/kg; 50 mg/kg IVIg) in the MCAO models (n = 20). At 2 h after ischemic-reperfusion, the mice were administrated with vehicle, or MPC-n(IVIg) (100 or 50 mg/kg). All experiments were repeated independently three times. All data are presented as the mean ± S.D. ns, nonsignificant, *P < 0.05, **P < 0.01, or ***P < 0.001.

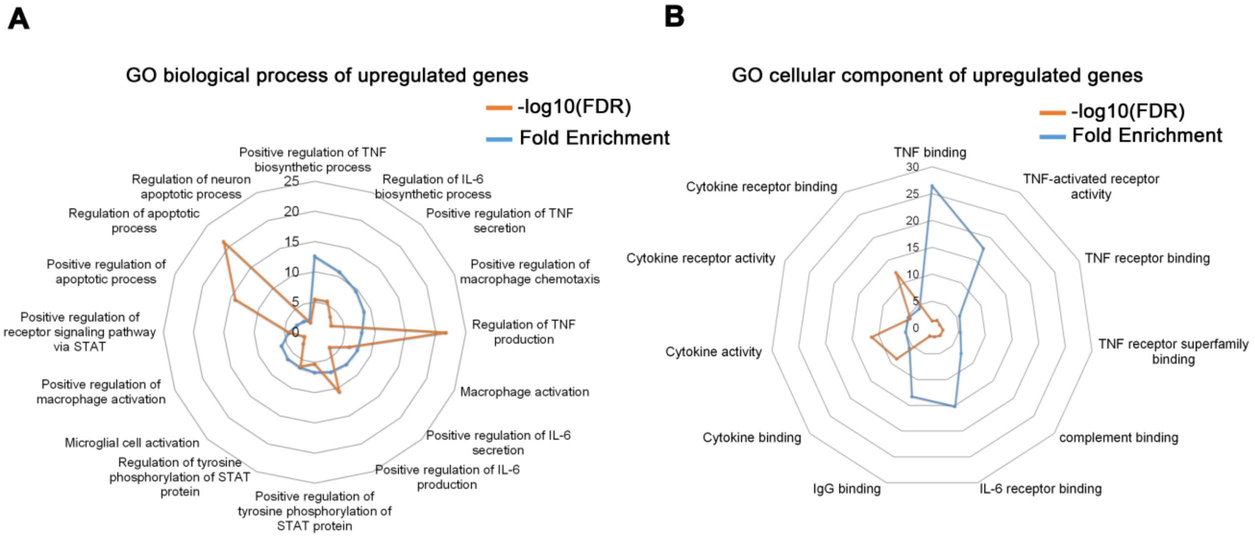

Figure S14. Acute inflammatory cascades initiated after stroke.

A) GO biological process of the upregulated genes in the MCAO group revealed the activation of production and secretion of TNF and IL-6, activation of monocytes, and regulation of the apoptotic process.

B) GO molecular function of the upregulated genes in the MCAO group associated with TNF, complement, IL-6, and IgG.

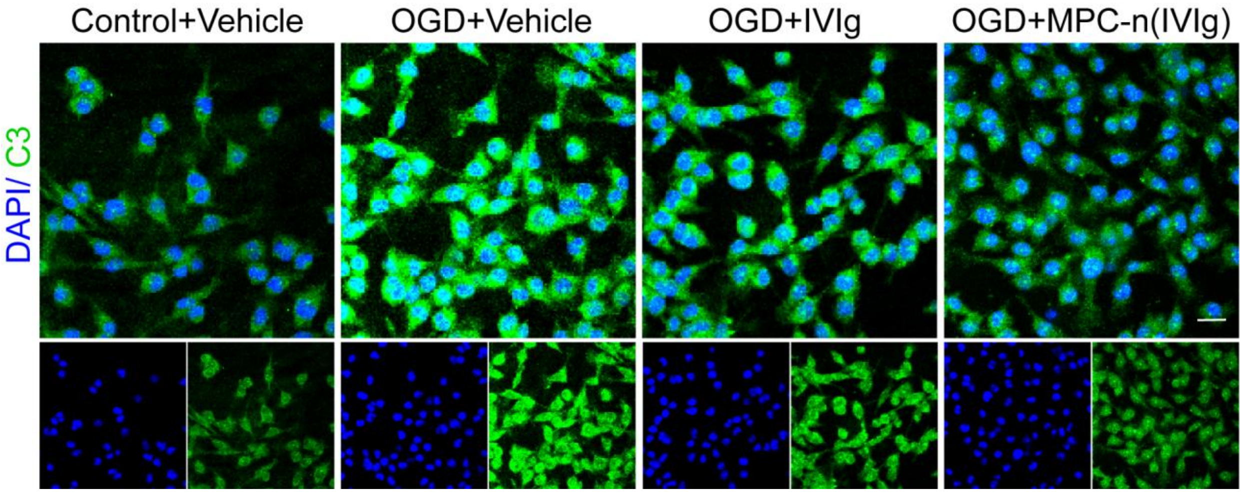

Figure S15. MPC-n(IVIg) suppresses C3 after OGD in BV-2 cells.

BV-2 cells were subjected to OGD for 12 h and incubated with vehicle, free IVIg, or MPC-n(IVIg) (10 mg/mL) for 24 h before immunofluorescence. BV-2 cells under normoxic conditions were the control. DAPI (blue), and C3 (green). Scale bar = 20 μm. All experiments were repeated independently three times.

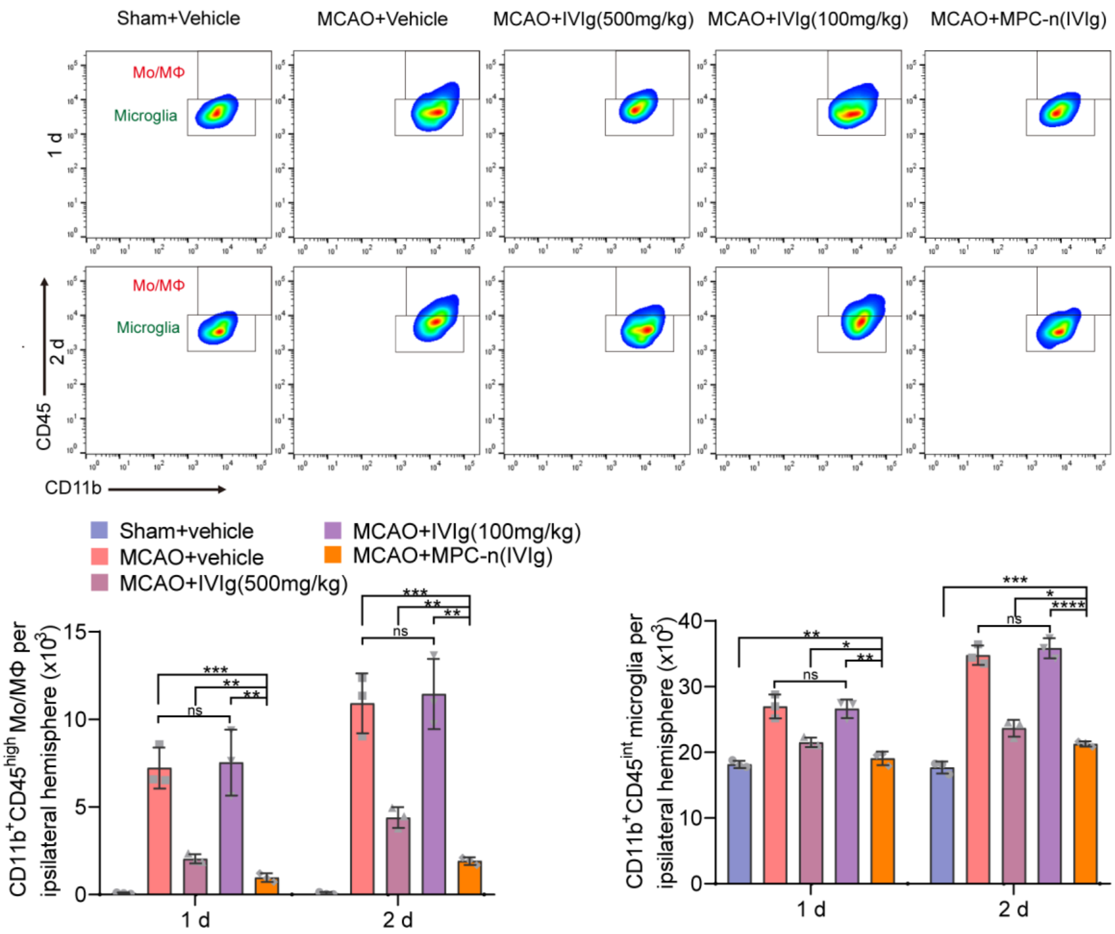

Figure S16. Mo/MΦ and microglia in the ischemic penumbra at 1 or 2 d after injection.

Representative plots of CD11b^{+} CD45^{int} microglia and CD11b $^{+}$ CD45^{hi} Mo/MΦ in the ischemic penumbra at 1 or 2 d after injection (n = 3).

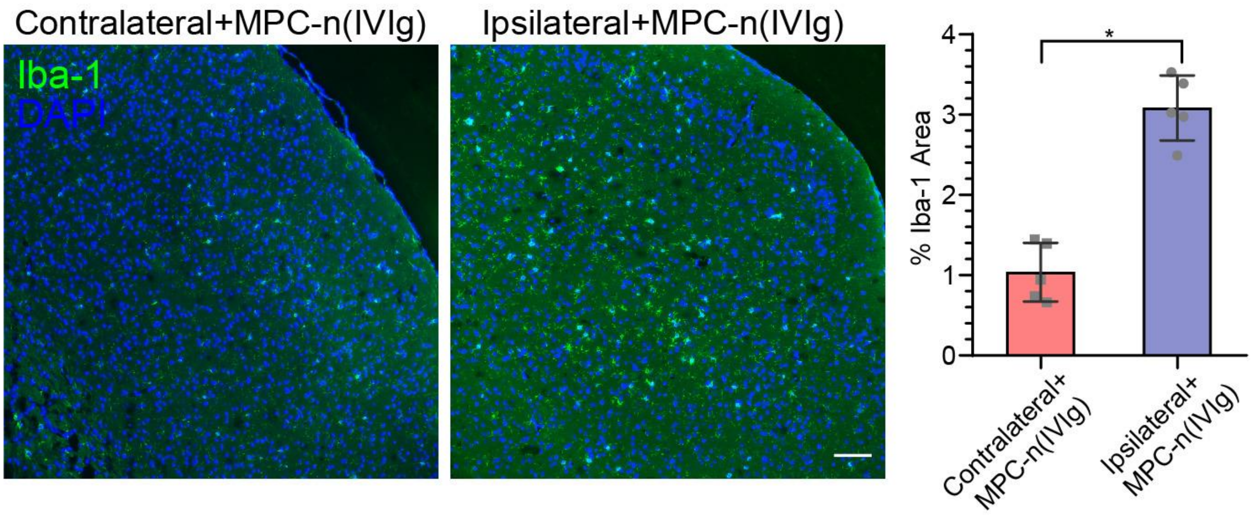

Figure S17. MPC-n(IVIg) does not completely block microglial activation in the ipsilateral hemisphere in MCAO models.

Fluorescence images of microglia around the ischemic penumbra 3 d post-injection. Iba-1 (green) and DAPI (blue) (n = 5). Scale bar = 20 μm. All experiments were repeated independently three times. All data are presented as the mean ± S.D. ns, nonsignificant, *P < 0.05, **P < 0.01, or ***P < 0.001.

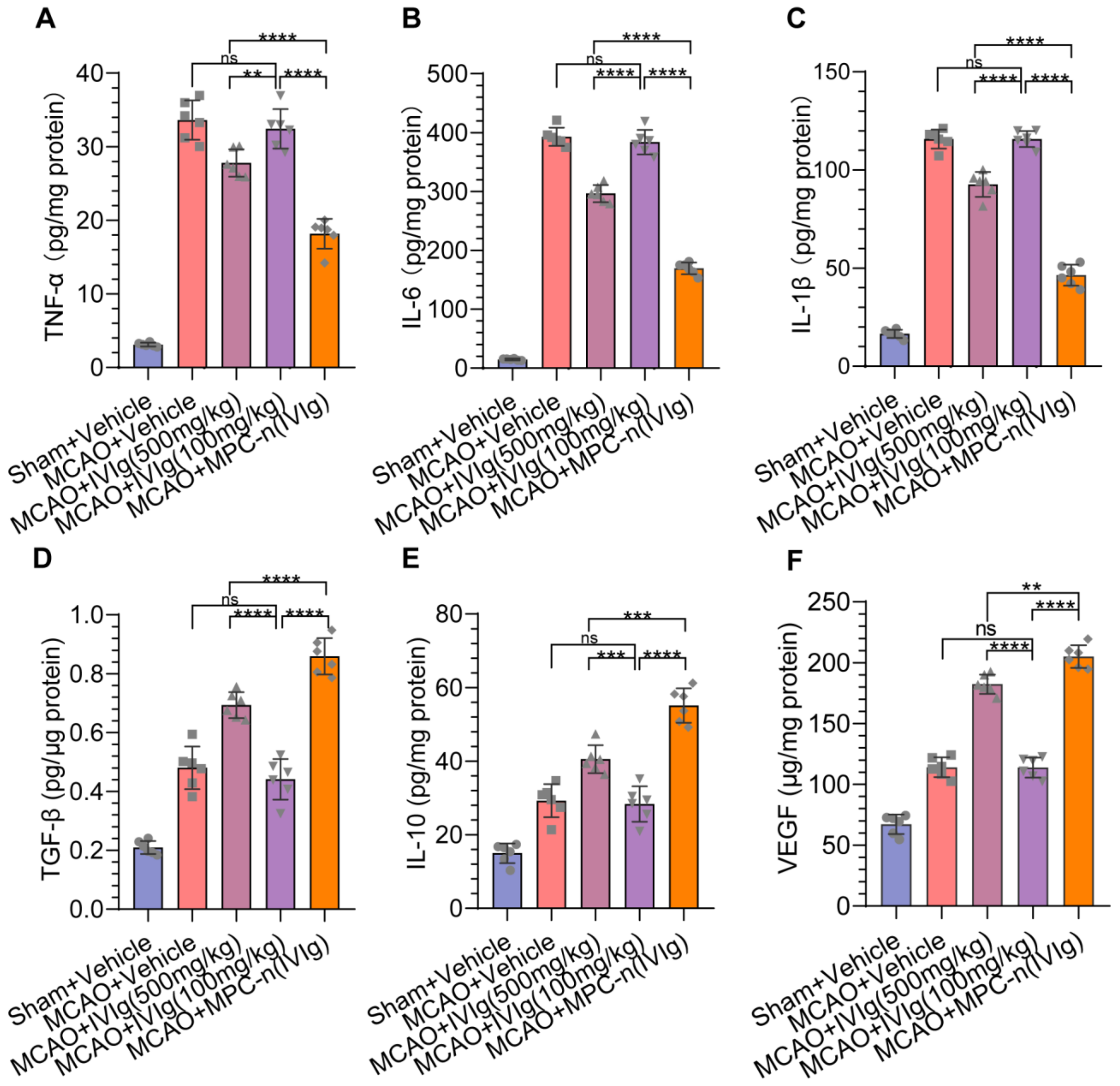

Figure S18. MPC-n(IVIg) decreases inflammation and enhances anti-inflammatory cytokines and VEGF in the brain tissue.

A-F) MCAO mice were injected with vehicle, free IVIg (100 and 500 mg/kg), and MPC-n(IVIg) (100 mg/kg). The inflammatory (TNF-α, IL-6, and IL-1β) and anti-inflammatory cytokines (TGF-β, IL-10, and VEGF) were measured in the ischemic hemispheres. ELISA was used to quantify the inflammatory (TNF-α, IL-6, and IL-1β), anti-inflammatory cytokines (TGF-β, and IL-10), and VEGF in the brain homogenates of the ipsilateral hemisphere (left brain, n = 6). All

experiments were repeated independently three times. All data are presented as the mean ± S.D. ns, nonsignificant, *P < 0.05, **P < 0.01, or ***P < 0.001.

Table S1 Detailed description of reagents used in this study.

REAGENT or RESOURCE	SOURCE	IDENTIFIER	DILUTION and APPLICATION
**Antibody/Fluorophore**			
Rabbit anti-NeuN	Abcam	Cat#ab177487	1:200, IF
Mouse anti-NeuN	Millipore	Cat#MAB337	1:400, IF
Mouse anti-MAP-2	Immunoway	Cat#YM1061	1:200, IF
Rabbit anti-GFAP	Cell Signaling Technology	Cat#80788S	1:200, IF
Rabbit anti-Iba-1	Cell Signaling Technology	Cat#17198S	1:200, IF
Mouse anti-CD31	Abcam	Cat#ab24590	1:200, IF
Rabbit anti-ZO-1	ThermoFisher	Cat#61-7300	1:200, IF
Mouse anti-ChT1	Millipore	Cat#MAB5514	1:200, IF
Rabbit anti-HIF-1α	Cell Signaling Technology	Cat#59370S	1:1000, Western blot
Rabbit anti-β-actin	Cell Signaling Technology	Cat#4970S	1:1000, Western blot
Rabbit anti-Complement C3	ThermoFisher	Cat#PA5-21349	1:200, IF
Goat anti -CD206	ThermoFisher	PA5-46994	1:200, IF
FITC anti-mouse CD86	Biolegend	105006	1:200, Flow cytometry
PerCP/Cyanine5.5 anti-mouse CD45 Antibody	Biolegend	103132	1:200, IF and Flow cytometry
FITC anti-mouse/human CD11b Antibody	Biolegend	101206	1:200, IF and Flow cytometry
Alexa Fluor^{TM} 594 phalloidin	Invitrogen	Cat#1906348	1:200, IF
Alexa Fluor^{TM} 633 goat anti-mouse IgG (H+L)	Invitrogen	A21052	1:200, IF
Alexa Fluor^{TM} 594 goat anti-mouse IgG (H+L)	Invitrogen	A11032	1:200, IF
Alexa Fluor^{TM} 488 donkey anti-mouse IgG (H+L)	Invitrogen	A21202	1:200, IF
Alexa Fluor^{TM} 488 goat anti-rabbit IgG (H+L)	Invitrogen	A11008	1:200, IF
Alexa Fluor^{TM} 594 donkey anti-rabbit IgG (H+L)	Invitrogen	A21207	1:200, IF
Alexa Fluor^{TM} 594 donkey anti-goat IgG (H+L)	Invitrogen	A11058	1:200, IF

**Critical Commercial Assays**		
Human IgG ELISA Kit	Abcam	ab195215
Mouse IL-10 Quantikine ELISA Kit	LanpaiBio	LP-M03457
Mouse/Rat/Porcine/Canine TGF-beta 1 Quantikine ELISA Kit	LanpaiBio	LP-M04110
Mouse TNF-α Quantikine ELISA Kit	Immunoway	KE1419
Mouse IL-6 Quantikine ELISA Kit	Immunoway	KE1418
Mouse IL-1β Quantikine ELISA Kit	Immunoway	KE1416
Mouse Complement C3b ELISA Kit	LanpaiBio	LP-M04515
Mouse VEGF ELISA Kit	LanpaiBio	LP-M03494
TUNEL Apoptosis Detection Kit (FITC)	YEASEN	40306ES20
Cell Counting Kit-8	MEC	HY-K0301-3000T
Annexin V-FITC/7AAD Apoptosis Detection Kit	BestBio	BB-41012-50T

Table S2. The sequences of siRNAs targeting ChT1 and HIF-1 α.

**Target**	**Catalog**	**Sequence**
siRNA (NC)	siRNA#1	5′-TTCTCCGAACGTGTCACGTTT-3′
mus-Slc5a7 (ChT1)	siRNA#1	5′-GGGATATGTGACTATGTTAGA-3′
	siRNA#2	5′–AGACCATTCTAGTCAGAAATG-3′
	siRNA#3	5′-GGATATTCTCTGAGTCTAATT-3′
mus-HIF1-α	siRNA#1	5′-CAGAGACGAAGGACAATAAAG-3′
	siRNA#2	5′-GATGGAAGCACTAGACAAAGT-3′
	siRNA#3	5′-CCATGATATGTTTACTAAAGG-3′

Table S3. The primers used for qPCR.

**Target**		**Sequence (5′-3′)**
β-actin mRNA	Forward	GGCTGTATTCCCCTCCATCG
	Reverse	CCAGTTGGTAACAATGCCATGT
ChT1 mRNA	Forward	GTGACGCAAAGAAGATTTGATGAAG
	Reverse	TCTGGGTTGCCGCTGTTTTT
CD86 mRNA	Forward	TCTCCACGGAAACAGCATCT
	Reverse	CTTACGGAAGCACCCATGAT

Table S4. Effects of MPC-n(IVIg) (100 mg/kg) on cumulative mortality in post-stroke mice.

Timepoint (day)	Sham+Vehicle, number dead (%) N at baseline	MCAO+Vehicle, number dead (%)	MCAO+IVIg (500 mg/Kg), number dead (%)	MCAO+IVIg (100 mg/Kg), number dead (%)	MCAO+MPC-n(IVIg), number dead (%)
	25	25	25	25	25
1	0 (0)	5 (20)	1 (4)	4 (16)	0 (0)
2	0 (0)	8 (32)	2 (8)	8 (32)	0 (0)
3	0 (0)	11 (44)	3 (12)	12 (48)	0 (0)
4	0 (0)	15 (60)	5 (20)	16 (64)	1 (4)
5	0 (0)	15 (60)	6 (24)	16 (64)	1 (4)
6	0 (0)	15 (60)	6 (24)	16 (64)	1 (4)
7	0 (0)	15 (60)	6 (24)	16 (64)	1 (4)
8	0 (0)	15 (60)	6 (24)	16 (64)	1 (4)
9	0 (0)	15 (60)	6 (24)	16 (64)	1 (4)
10	0 (0)	15 (60)	6 (24)	16 (64)	1 (4)

Mortality was monitored for 10 days after injection. The cumulative numbers of dead animals at each time-point are shown with the rate (percentage) of death being shown in parenthesis.
